# Entropy Methods on Finding Optimal Linear Combinations with an Application to Biomarkers

**DOI:** 10.3390/e27090985

**Published:** 2025-09-21

**Authors:** Mehmet Sinan İyisoy, Pınar Özdemir

**Affiliations:** 1 Department of Medical Education and Informatics, Necmettin Erbakan University, Konya 42090, Turkey; 2 Department of Biostatistics, Hacettepe University, Ankara 06100, Turkey; pbc.ozdemir@gmail.com

**Keywords:** linear combination, binary outcome, biomarkers, 62B10, 94A15

## Abstract

Identifying an optimal linear combination of continuous variables is a key objective in various fields of research, such as medicine. This manuscript explores the use of information-theoretical approaches used to establish these linear combinations. Coefficients obtained from logistic regression can be used to construct such a linear combination, and this approach has been commonly adopted in the literature for comparison purposes. The main contribution of this work is to propose novel ways of determining these linear combination coefficients by optimizing information-theoretical objective functions. Biomarkers are usually continuous measurements utilized to diagnose if a patient has the underlying disease. Certain disease contexts may lack high diagnostic power biomarkers, making their optimal combination a critical area of interest. We apply the above-mentioned novel methods to the problem of a combination of biomarkers. We assess the performance of our proposed methods against combinations derived from logistic regression coefficients, by comparing area under the ROC curve (AUC) values and other metrics in a broad simulation and a real life data application.

## 1. Introduction

Information theoretical concepts are inherently and deeply linked to statistical principles. These concepts play a crucial role in the processes of data analysis and modeling. The rich set of tools information theory offers focus on quantifying uncertainty in a random variable, divergence, dependence, and information gain.

They facilitate the understanding of how data can be utilized more efficiently, which variables provide greater informational value, and how uncertainty within the data can be quantified. They are used in areas like model selection, estimation, hypothesis testing, learning, and decision-making.

By integrating information-theoretic objectives into statistical inference and learning, researchers can develop models that are both interpretable and data-efficient, making these methods especially valuable in high-dimensional and data-scarce domains such as bioinformatics, neuroscience, and medical diagnostics.

To name a few, tools and notions from information theory such as Shannon entropy, Kullback–Leibler divergence, and mutual information provide rigorous means for assessing the complexity and informativeness of models and data. These measures are central to a wide range of statistical applications, including model selection (e.g., through criteria like AIC, Akaike’s Information Criteria and MDL, Mininum Description Length), feature selection, clustering, density estimation, and hypothesis testing.

Additionally, methods based on the principle of maximum entropy allow for the estimation of probability distributions under partial information, yielding minimally biased solutions constrained only by known data characteristics. Cross-entropy and information gain are widely used in classification tasks and decision tree algorithms, respectively, to optimize predictive performance.

To define the core problem addressed in this manuscript, consider a given matrix *X* of dimension nxd with continuous individual predictors $x_{1},x_{2},\ldots ,x_{d}$ being its columns and a given binary outcome vector *y* of 0/1. Our aim is to find a coefficient vector *w* such that the linear combination Xw would be a better predictor of *y* than all individual columns $x_{1},x_{2},\ldots ,x_{d}$ of *X*.

It is well known that logistic regression maximizes the logarithm of the likelihood function

$$l\left(\beta_{0},\beta_{1},\beta_{2},\ldots ,\beta_{d}\right)=\prod_{i=1}^{n}P\left(y_{i}|\;X_{i},\beta_{0},\beta_{1},\beta_{2},\ldots ,\beta_{d}\right)=\prod_{i=1}^{n}{p(X_{i})}^{y_{i}}{\left[1-p(X_{i})\right]}^{1-y_{i}}$$

having $p\left(X_{i}\right)=P\left(y=1|\;X_{i},\beta \right)=\sigma \left(X_{i}\beta \right)$ to estimate coefficients vector β where $\sigma \left(x\right)=\frac{1}{1+e^{-x}}$ is the sigmoid function, *n* is the number of samples, and $X_{i}$ is the $i^{th}$ row of *X*. After estimating coefficients vector β from logistic regression, one can easily select $w=(\beta_{1},\beta_{2},\ldots ,\beta_{d})$ as a candidate coefficient vector to be used for linear combination of individual predictors.

The novel approaches we will exhibit in the following pages utilize information-theoretical principles for the determination of coefficient vector *w*. Section 2 reviews previous methods used for linear combinations of biomarkers, all of which are non-information-theoretic in nature. Section 3 is mainly for the details of our proposed methods. Section 4 is devoted to details and results of the simulation, and Section 5 showcases an application to real-world data.

## 2. Previous Methods Used in Linear Combinations of Biomarkers

A biomarker is typically employed as a diagnostic or evaluative tool in medical research. Identifying a single ideal biomarker that offers both high sensitivity and specificity is challenging, particularly when high specificity is needed for population screening. An effective alternative is the combination of multiple biomarkers, which can deliver superior performance compared to using just one biomarker.

Especially in cases where there is more than one weak biomarker, it is important to use them in combination [1]. Combining biomarkers has several advantages such as increased diagnostic accuracy, better prediction of disease course and prognosis, and the development of personalized medical applications.

Numerous studies in literature employ ROC (Receiver Operating Characteristic) analysis to assess the performance of combined biomarkers developed through various methodologies. Those in binary classification focused on maximizing various non-parametric estimates of the area under the ROC curve (AUC) or the Mann–Whitney U statistic to derive the optimal linear combination of single biomarkers.

AUC is an essential performance indicator of binary models. AUC values show the model’s ability to distinguish between two classes (0/1) of the binary outcome (discriminative ability). High AUC values indicate better and more accurate discrimination.

The authors in [2] used Fisher’s discriminant functions under multivariate normality and (non)proportional covariance settings to produce a linear combination which maximizes AUC. Later, the authors in [3] enhanced and investigated some other properties of these linear combinations and proposed alternative combinations.

The authors in [4] proposed a distribution-free rank based approach for deriving linear combinations of two biomarkers that maximize the area or partial area under the emprical ROC curve. They compared its performance to those obtained from optimizing logistic likelihood function and linear discriminant analysis. They claimed that linear discriminant analysis optimizes AUC when multivariate normality was achieved. They provided further insights such as selecting emprical AUC as an objective function may yield better performances than selecting a logistic likelihood function in another publication [5].

The nonparametric Min–Max procedure proposed in [6] is claimed to be more robust to distributional assumptions, is easier to compute, and has better performance. The methodology proposed in [7] extended combination approaches to the problem where outcome is not dichotomous but ordinal with more than two categories. They proposed two new methods and compared their performances with existing methods.

In the study [1], they compared existing methods where the number of biomarkers are large, biomarkers are weak, and the number of observations are not an order of magnitude greater than the number of biomarkers. They also proposed a new method for combinations.

Underlining certain inadequacies of present combination methods, the authors in [8] proposed a new kernel-based AUC optimization method and claimed that it outperformed the smoothed AUC method previously proposed in [9].

The authors in [10] propose a derivative-free black-box optimization technique, called Spherically Constrained Optimization Routine (SCOR), to identify optimal linear combinations where the outcome is ordinal. The method proposed in [11], Nonparametric Predictive Inference (NPI), examines the best linear combination of two biomarkers, where the dependency between two biomarkers is modeled using parametric copulas. Another copula-based combination method in [12] utilized different copulas for an optimal linear combination of two biomarkers with a binary outcome.

It is important to note that none of the preceding methods employed information-theoretical concepts in their methodologies.

## 3. Information Theoretical Methods for Linear Combinations

This section details maximum entropy, minimum cross entropy, minimum relative entropy, and maximum mutual information concepts.

### 3.1. Entropy Maximization MaxEnt

Having historical roots in physics, the maximum entropy principle is an approach to finding the distribution that maximizes the entropy of a probability distribution under certain constraints. It is proposed by Jaynes [13] as a general principle of inference and has been applied successfully to numerous fields. Maximizing entropy means choosing the distribution that reflects maximum uncertainty. Axiomatic derivation of maximum entropy and minimum cross entropy principle was done by [14]. The authors in [15] applied the maximum entropy principle to recover information from multinomial data.

Suppose we know that a system has a set of possible states $y_{k},k\in \left\{0,1\right\}$ with unknown probabilities $p_{ik}=P\left(Y=y_{k}|X\right)$ for i=1,2,…,n and we have a given matrix *X* of dimension nxd and a binary vector *y* of dimension *n*. We want to find a coefficient vector *w* of dimension *d* so that vector Xw would be a better predictor of *Y*.

The maximum entropy principle explains how to select a certain distribution $\hat{p}$ among different possible distributions that satisfy our constraints. We treat $p_{ik}$ as variables when finding the maximum entropy solution to our problem. Notice that, since we deal with binary vector *y* and $p_{i0}=1-p_{i1}$, solving for $p_{i1}=P\left(Y=y_{1}|X\right)$ will suffice and reduce the complexity of the problem.

In mathematical terms, we want to find $\hat{p}$ that maximizes H(p):

$$\hat{p}=\underset{p}{\arg\max}H\left(p\right)=\underset{p}{\arg\max}-\sum_{i=1}^{n}\left(p_{i0}\log p_{i0}+p_{i1}\log p_{i1}\right)$$

Replacing $p_{i0}=1-p_{i1}$, this would become

$$\hat{p}=\underset{p}{\arg\max}-\sum_{i=1}^{n}\left[\left(1-p_{i1}\right)\log\left(1-p_{i1}\right)+p_{i1}\log p_{i1}\right]$$

We impose the following constraints to fit the data:

(1) $$\sum_{i=1}^{n}X_{ij}y_{i}=\sum_{i=1}^{n}X_{ij}p_{i1},\;\;j=1,2,\ldots ,d,$$

and

(2) $$\sum_{k=1}^{2}p_{ik}=1$$

where $\sum_{i=1}^{n}X_{ij}y_{i}$ values are empirical expectations obtained from data and d+1<<n. Since, in general, we have many more observations than predictors, *n* is much larger than d+1. We can relax the second constraint (2) to $0\leq p_{i1}\leq 1$ since $p_{i0}$ values can be easily obtained by $p_{i0}=1-p_{i1}$. Hence, the final optimization problem becomes

$$\hat{p}=\underset{p}{\arg\max}-\sum_{i=1}^{n}\left[\left(1-p_{i1}\right){\log{(1-p}_{i1})+p}_{i1}\log p_{i1}\right]$$

under the constraints

$$\sum_{i=1}^{n}X_{ij}y_{i}=\sum_{i=1}^{n}X_{ij}p_{i1},\;\;j=1,2,\ldots ,d$$

and

$$0\leq p_{i1}\leq 1.$$

This is a solvable convex optimization problem with linear constraints. After determining $\hat{p}$ namely $p_{i1}$ values, *w* still needs to be found. Since we based the problem on finding probabilities first rather than the coefficient vector *w* first, we need to glue these probabilities to our given matrix *X*. We use logits

$$l_{i}=log\frac{p_{i1}}{1-p_{i1}}$$

obtained from those probabilities and perform least squares estimation to equation Xw=l where *l* is the logits vector.

Other ways of estimating *w* without directly finding probabilities were possible, as we did below in cross entropy minimization. However, we deliberately focused here on this method to show that the method finds probabilities complying to the Maximum Entropy principle.

### 3.2. Cross-Entropy Minimization MinCrEnt

First proposed by Kullback [14], this principle incorporates cross entropy of two distributions to find the optimal solution.

Suppose we know that a system has a set of possible states $y_{k},k\in \left\{0,1\right\}$ with unknown probabilities $p_{ik}=P\left(Y=y_{k}|X\right)$ for i=1,2,…,n, as described in Section 3.1.

The principle states that, among different possible distributions *p* that satisfy certain constraints, one should select the one which minimizes the cross entropy with *q* where *q* is a known a priori distribution. In our case, we select *q* to be the empirical distribution of the outcome (namely, *Y*):

$$\hat{p}=\underset{p}{\arg\min}H\left(p,q\right)=\underset{p}{\arg\min}-\sum_{i=1}^{n}\left(p_{i0}\log q_{i0}+p_{i1}\log q_{i1}\right).$$

Again using $p_{i0}=1-p_{i1}$, we can simplify the objective function to

$$\hat{p}=\underset{p}{\arg\min}-\sum_{i=1}^{n}\left[\left(1-p_{i1}\right)\log\left(1-q_{i1}\right)+p_{i1}\log q_{i1}\right].$$

The objective function we obtained on right hand side indeed coincides with the negative of log-likelihood function from logistic regression. We know the distribution of $q_{i1}$ values a priori, but we do not know the $p_{i1}$ value. A natural and inevitable choice for getting $p_{i1}$ values is to use a transformation of given data matrix X with coefficient vector *w*. Thus, we define

$$p_{i1}=\sigma \left(\sum_{j=1}^{d}X_{ij}*w_{j}\right)$$

where σ is the sigmoid function. Hence, the optimization problem becomes

$$\hat{p}=\underset{p}{\arg\min}H\left(p,q\right)$$

$$=\underset{p}{\arg\min}-\left\{\sum_{i=1}^{n}\left[1-\sigma \left(\sum_{j=1}^{d}X_{ij}*w_{j}\right)\right]\log\left(1-q_{i1}\right)+\sigma \left(\sum_{j=1}^{d}X_{ij}*w_{j}\right)\log q_{i1}\right\}$$

where $q_{i1}$ values are known probabilities obtained from vector y.

We do not impose any further constraints here because use of sigmoid function confines the probabilities between 0 and 1, and we already made use of X. This is an unconstrained convex optimization problem that can easily be solved.

### 3.3. Relative Entropy Minimization (MinRelEnt)

Relative entropy minimization is a technique less known and partly misidentified with cross entropy minimization [16]. Like cross entropy, relative entropy requires a reference distribution. We used the empirical distribution of *y* as a reference distribution *q* in cross entropy minimization, but we choose the uniform distribution $q_{i1}=0.5$ here. The optimization problem becomes

$$\hat{p}=\underset{p}{\arg\min}D\left(p∥q\right)=\underset{p}{\arg\min}\left\{-\sum_{i=1}^{n}\left(p_{i0}\log\frac{p_{i0}}{q_{i0}}+p_{i1}\log\frac{p_{i1}}{q_{i1}}\right)\right\}$$

or

$$\hat{p}=\underset{p}{\arg\min}\left\{-\sum_{i=1}^{n}\left(\left(1-p_{i1}\right)\log\frac{1-p_{i1}}{{1-q}_{i1}}+p_{i1}\log\frac{p_{i1}}{q_{i1}}\right)\right\}$$

As we did in entropy maximization, we use the same constraint on empirical expectations to determine probabilities

$$\sum_{i=1}^{n}X_{ij}y_{i}=\sum_{i=1}^{n}X_{ij}p_{i1},\;\;j=1,2,\ldots ,d$$

and for probabilities we set

$$0\leq \;p_{i1}\leq 1.$$

This is also a convex optimization problem with linear constraints. After finding probabilities, we transform them to logits and solve Xw=l where *l* is the logits vector using least squares as we did for entropy maximization. It is worth noting that alternative choices for $q_{i1}$ are possible, such as the empirical distribution of *y* or incorporating any other prior belief about *y*.

### 3.4. Mutual Information Maximization MaxMutInf

As was previously done in a similar but not the same setting by Faivishevsky and Goldberger [17], we estimate coefficient vector *w* through mutual information maximization. We use the following identity for mutual information:

$$I_{mean}\left(X;Y\right)=H_{mean}\left(X\right)-H_{mean}\left(X|Y\right)$$

where *X* is the predictor matrix, *Y* is the emprical distribution of outcome vector *y* in our problem, and $I_{mean}$ is the mutual information estimator defined by Faivishevsky and Goldberger. Since *Y* is categorical, we deal with conditional entropies corresponding to states of $Y,y_{k}\left(k\in \left\{0,1\right\}\right)$:

$$H_{mean}\left(X|Y\right)=\sum_{k\in \left\{0,1\right\}}p\left(Y=y_{k}\right)H_{mean}\left(X|\;Y=y_{k}\right)$$

where $H_{mean}\left(X|Y=y_{k}\right)$ is the entropy of *X* restricted to values where *Y* takes the value $y_{k}$ (also called in-class entropy). Hence, the optimization problem becomes

$$\hat{w}=\arg\max_{w}I_{mean}\left(Xw;Y\right)=\arg\max_{w}\left(H_{mean}\left(Xw\right)-\sum_{k\in \left\{0,1\right\}}p\left(Y=y_{k}\right)H_{mean}\left(Xw|\;Y=y_{k}\right)\right)$$

or

$$\hat{w}=\arg\max_{w}\left(\frac{1}{n(n-1)}\sum_{i\neq j}\log{∥\left(x_{i}-x_{j}\right)w∥}^{2}-\sum_{k\in \left\{0,1\right\}}\frac{p\left(Y=y_{k}\right)}{n_{k}\left(n_{k}-1\right)}\sum_{i_{k}\neq j_{k}}\log{∥\left(x_{ik}-x_{jk}\right)w∥}^{2}\right)$$

where $n_{k}$ is the number of observations having class value $y_{k}$. It is easy to see that $\sum_{k\in \{0,1\}}n_{k}=n$. The smoothness of MeanNN entropy estimator enables its gradient analytically computed. Therefore, $I_{mean}\left(Xw,Y\right)$ with respect to *w* becomes

$$\frac{\partial I_{mean}}{\partial w}=\frac{2}{n(n-1)}\sum_{i\neq j}\frac{\left(x_{i}-x_{j}\right){\left(x_{i}-x_{j}\right)}^{T}w}{{∥\left(x_{i}-x_{j}\right)w∥}^{2}}-\sum_{k\in \{0,1\}}\frac{2p\left(Y=y_{k}\right)}{n_{k}\left(n_{k}-1\right)}\sum_{i_{k}\neq j_{k}}\frac{\left(x_{ik}-x_{jk}\right){\left(x_{ik}-x_{jk}\right)}^{T}w}{{∥\left(x_{i}-x_{j}\right)w∥}^{2}}$$

Since the gradient of mutual information is achieved, we use gradient ascent method to maximize mutual information.

## 4. Simulation

An extensive simulation study was conducted to compare the efficiencies of those methods on combination of continuous variables (or biomarkers). Imitating but also enriching the one performed previously in [1] for comparison purposes, we considered normal, gamma, and beta distributions under different settings.

In all settings, we assumed mean values $\mu_{0}=0$ corresponding to class $y_{0}$. For class $y_{1}$, we set mean values as $\mu_{1}=0.4\frac{id}{n-1}$, where *d* denotes the number of predictors and 0≤i≤d−1. As a result, the mean values for $y_{0,1}$ classes were evenly spaced and dependent on the number of predictors *d*. Candidate biomarkers generated through this approach, typically have AUC values between 0.5≤AUC≤0.75, most commonly falling below 0.7.

We used multivariate normal variables with equal and not equal covariance structures to generate normal, beta, and gamma variates using normal copula and inverse transform sampling. When covariances are equal, we fixed $\Sigma_{0}=\Sigma_{1}=\left(1-\gamma \right)I_{dxd}+\gamma J_{dxd}$ where I is the identity matrix, J is a matrix of all 1 s, and γ=0.15. For unequal covariance, we set $\Sigma_{0}=0.9I_{dxd}+0.1J_{dxd}$ keeping $\Sigma_{1}=\left(1-\gamma \right)I_{dxd}+\gamma J_{dxd}$ unchanged.

For each setting, 1000 datasets were generated and randomly divided into two sets of equal size. One set was used for training and the other for testing. Coefficient estimates obtained from training datasets were recorded for each method. Using the test datasets, linear combinations were computed based on the previously estimated coefficients, and corresponding performance metrics were evaluated. These metrics included AUC, Area Under the Precision–Recall Curve (AUPRC), and Matthews Correlation Coefficient (MCC). In addition to mean values, 95% confidence intervals and median values were reported for each metric.

Sample sizes were set to n=25, 100, 200, and the number of predictors was varied across d=3,5,10,15. Unlike the referenced simulation study, we also examined scenarios with unequal class allocation, specifically cases where $n_{0}=2n_{1}$. In the unequal class allocation scenarios, the sample sizes for the y=1 class were adjusted to $n_{1}=12,50,100$. Table 1, Table 2 and Table 3 present AUC values obtained from the simulation results. Additional results including those for AUPRC, MCC, and all unequal allocation cases are provided in the Supplementary Material.

All simulations were conducted using Julia version 1.11.0-rc3. We utilized the Ipopt.jl package version 1.11.0 for nonlinear constrained optimization in entropy-based methods, the GLM.jl package version 1.9.0 for logistic regression, and custom gradient ascent code for the mutual information maximization method.

The code used in the simulations will be released as public Julia and R packages in future publications. Interested researchers may contact the author for access.

## 5. Application to Real-Life Data

We used publicly available Wisconsin datasets for the prognosis [18] and diagnosis [19] of breast cancer. There are 30 continuous predictors in each dataset to be used for the prediction of either prognosis or diagnosis of breast cancer indicated 0/1.

The prognostic dataset has 198 observations while the diagnostic has 569. Frequencies of $y_{1}$ class are 47/198 and 212/569 in those datasets. We considered the first 5 predictors in each dataset for our calculations, namely the variables radius_mean, texture_mean, perimeter_mean, area_mean, and smoothness_mean.

Predictive ability of these variables are given in Table 4. As can be seen from the Table 4, those predictors have a very low predictive ability for the prognosis of breast cancer, but a high ability for diagnosis.

We performed logistic regression and entropy optimization techniques described in Section 3 and gathered coefficients shown in Table 5 and Table 6, representing candidate linear combinations for both datasets.

Finally, calculating linear combinations using those coefficients, we obtained AUC, AUPRC, and MCC values given in Table 7 and Table 8. Our methods yielded metric values very similar to those derived from logistic regression.

## 6. Discussion and Conclusions

The primary objective of this study was to explore how information-theoretical methods can be applied to construct linear combinations of continuous variables in the context of a binary classification problem.

We addressed this question by introducing four distinct approaches (MaxEnt, MinCrEnt, MinRelEnt, and MaxMutInf), each grounded in fundamental principles of information theory.

We believe that identifying and formalizing these approaches may guide future research directions by drawing greater attention to information-theoretical concepts and encouraging their broader application in this problem setting. This study also represents the first systematic evaluation of information-theoretic approaches applied in this setting.

Earlier methods were often constrained by strong distributional assumptions about biomarkers, such as normality and equal or proportional covariance structures. Additionally, the number of predictors they could handle was typically limited to just two.

More recent approaches have relaxed these assumptions, allowing for the inclusion of a larger number of biomarkers in linear combinations. However, most of these methods rely on performance metrics like sensitivity, specific segments of the ROC curve, or the AUC (Area Under the Curve) as the optimization objective. Some later methods extended their applicability to multi-class classification problems using metrics such as the Volume Under the Surface (VUS) or the Hypervolume Under the Manifold (HUM).

Among these, the SCOR algorithm [10] and the method proposed in [1] have shown promising results in maximizing the AUC or HUM objective.

Notably, methods established in [11,13] distinguish themselves by incorporating copulas into the picture, representing the first known use of copulas in the context of biomarker combination. Despite this innovation, they currently remain limited to handling only two biomarkers.

An important distinction of our proposed methods is that, whereas previous methods primarily aimed to maximize a metric such as the AUC value, the proposed methods in this article rely solely on information-theoretic criteria and never incorporated AUC or any other metric as an optimization objective.

As another distinction, unlike many previous studies that relied on a single evaluation metric, our assessment of model performance employed a comprehensive set of three metrics: AUC, AUPRC, and MCC. This multi-faceted approach provides a more robust and nuanced comparison of model performance across different aspects of classification quality.

The proposed methods here are straightforward to apply in binary classification problems or to be extended into a multiclass setting. These methods are computationally simpler than most existing approaches, with the exception of MaxMutInf. Due to the need to compute complex gradient functions when optimizing mutual information, MaxMutInf is relatively more challenging to implement. Extending our methods into classification problems involving more than two outcome levels may represent a valuable direction for future research.

The three entropy-based methods (MaxEnt, MinCrEnt, and MinRelEnt) demonstrated test performances that were consistently comparable to those derived from logistic regression, across all simulation conditions and real data applications. The MaxMutInf method yielded slightly greater test AUC, AUPRC, and MCC values compared to the other approaches, particularly in simulation settings involving Beta and Gamma distributed data. The differences were more pronounced in simulations with smaller sample sizes. However, in some settings involving normally distributed data, the MaxMutInf method produced test AUC and AUPRC metrics that were comparable or even marginally lower than those of other methods.

The Maximum Mutual Information method appeared more robust to distributional asymmetries, as well as to smaller and unequal sample sizes, except in a few settings involving normally distributed variables. Exploring and implementing different differential entropy estimators beyond the Kozachenko–Leonenko entropy estimator may be a future research direction that could potentially reveal more insights into the performance of Mutual Information Maximization.

## Figures and Tables

**Table 1:** Mean AUC values with 95% confidence intervals obtained for multivariate Normal distributions.

			LogRes		MaxMutInf		MaxEnt		MinRelEnt		MinCrEnt	
**n**	**d**	**Covariance**	**Mean (95%CI)**	**Median**	**Mean (95%CI)**	**Median**	**Mean (95%CI)**	**Median**	**Mean (95%CI)**	**Median**	**Mean (95%CI)**	**Median**
25	3	Equal	0.615 (0.545,0.673)	0.597	0.617 (0.547,0.669)	0.603	0.615 (0.545,0.673)	0.597	0.615 (0.545,0.673)	0.597	0.615 (0.545,0.673)	0.597
25	5	Equal	0.619 (0.551,0.673)	0.604	0.625 (0.557,0.686)	0.609	0.62 (0.552,0.673)	0.608	0.62 (0.552,0.673)	0.608	0.62 (0.552,0.673)	0.607
25	10	Equal	0.613 (0.545,0.662)	0.597	0.634 (0.555,0.693)	0.622	0.612 (0.545,0.667)	0.597	0.612 (0.545,0.667)	0.597	0.61 (0.545,0.662)	0.591
25	15	Equal	0.614 (0.551,0.662)	0.597	0.634 (0.558,0.691)	0.622	0.612 (0.545,0.667)	0.596	0.612 (0.545,0.667)	0.596	0.611 (0.545,0.66)	0.596
100	3	Equal	0.599 (0.555,0.639)	0.598	0.585 (0.546,0.619)	0.580	0.599 (0.556,0.638)	0.599	0.599 (0.556,0.638)	0.599	0.599 (0.556,0.638)	0.599
100	5	Equal	0.601 (0.556,0.642)	0.596	0.593 (0.55,0.631)	0.591	0.601 (0.557,0.643)	0.597	0.601 (0.557,0.643)	0.597	0.601 (0.557,0.643)	0.597
100	10	Equal	0.612 (0.571,0.652)	0.610	0.608 (0.569,0.646)	0.606	0.612 (0.572,0.653)	0.612	0.612 (0.572,0.653)	0.612	0.612 (0.572,0.653)	0.612
100	15	Equal	0.617 (0.575,0.658)	0.617	0.616 (0.575,0.653)	0.615	0.618 (0.575,0.657)	0.617	0.618 (0.575,0.657)	0.617	0.618 (0.575,0.657)	0.617
200	3	Equal	0.607 (0.577,0.636)	0.608	0.582 (0.547,0.612)	0.580	0.607 (0.578,0.636)	0.607	0.607 (0.578,0.636)	0.607	0.607 (0.578,0.636)	0.607
200	5	Equal	0.614 (0.587,0.642)	0.614	0.592 (0.562,0.62)	0.592	0.615 (0.587,0.642)	0.615	0.615 (0.587,0.642)	0.615	0.615 (0.587,0.642)	0.615
200	10	Equal	0.633 (0.605,0.664)	0.635	0.61 (0.582,0.638)	0.611	0.634 (0.605,0.664)	0.636	0.634 (0.605,0.664)	0.636	0.634 (0.605,0.664)	0.636
200	15	Equal	0.641 (0.612,0.673)	0.643	0.616 (0.588,0.646)	0.616	0.642 (0.613,0.673)	0.644	0.642 (0.613,0.673)	0.644	0.642 (0.613,0.673)	0.644
25	3	Not Equal	0.615 (0.545,0.669)	0.597	0.617 (0.547,0.673)	0.603	0.614 (0.545,0.673)	0.597	0.614 (0.545,0.673)	0.597	0.614 (0.545,0.673)	0.597
25	5	Not Equal	0.62 (0.551,0.673)	0.604	0.626 (0.558,0.687)	0.610	0.621 (0.551,0.673)	0.609	0.621 (0.551,0.673)	0.609	0.621 (0.551,0.673)	0.609
25	10	Not Equal	0.615 (0.549,0.667)	0.603	0.637 (0.564,0.7)	0.623	0.614 (0.547,0.667)	0.603	0.614 (0.547,0.667)	0.603	0.612 (0.545,0.667)	0.596
25	15	Not Equal	0.615 (0.551,0.667)	0.603	0.638 (0.564,0.699)	0.623	0.613 (0.547,0.667)	0.597	0.613 (0.547,0.667)	0.597	0.611 (0.547,0.662)	0.596
100	3	Not Equal	0.599 (0.556,0.638)	0.600	0.586 (0.546,0.621)	0.582	0.599 (0.556,0.639)	0.599	0.599 (0.556,0.639)	0.599	0.599 (0.556,0.639)	0.599
100	5	Not Equal	0.602 (0.559,0.643)	0.599	0.595 (0.551,0.633)	0.593	0.602 (0.558,0.644)	0.600	0.602 (0.558,0.644)	0.600	0.602 (0.558,0.644)	0.600
100	10	Not Equal	0.614 (0.574,0.653)	0.614	0.613 (0.574,0.65)	0.611	0.615 (0.575,0.655)	0.615	0.615 (0.575,0.655)	0.615	0.615 (0.575,0.655)	0.615
100	15	Not Equal	0.62 (0.578,0.659)	0.620	0.621 (0.582,0.658)	0.621	0.621 (0.579,0.66)	0.622	0.621 (0.579,0.66)	0.622	0.621 (0.579,0.66)	0.622
200	3	Not Equal	0.607 (0.578,0.635)	0.607	0.583 (0.548,0.614)	0.581	0.607 (0.579,0.636)	0.607	0.607 (0.579,0.636)	0.607	0.607 (0.579,0.636)	0.607
200	5	Not Equal	0.616 (0.589,0.644)	0.615	0.594 (0.564,0.623)	0.594	0.616 (0.589,0.643)	0.615	0.616 (0.589,0.643)	0.615	0.616 (0.589,0.643)	0.615
200	10	Not Equal	0.636 (0.608,0.667)	0.637	0.615 (0.586,0.643)	0.617	0.637 (0.61,0.666)	0.638	0.637 (0.61,0.666)	0.638	0.637 (0.61,0.666)	0.638
200	15	Not Equal	0.644 (0.616,0.676)	0.646	0.623 (0.594,0.653)	0.622	0.645 (0.616,0.676)	0.647	0.645 (0.616,0.676)	0.647	0.645 (0.616,0.676)	0.647

Note: Statistics presented in this table were derived from test datasets described in Section 4.

**Table 2:** Mean AUC values with 95% confidence intervals obtained for Gamma distributions.

			LogRes		MaxMutInf		MaxEnt		MinRelEnt		MinCrEnt	
**n**	**d**	**Covariance**	**Mean (95%CI)**	**Median**	**Mean (95%CI)**	**Median**	**Mean (95%CI)**	**Median**	**Mean (95%CI)**	**Median**	**Mean (95%CI)**	**Median**
25	3	Equal	0.613 (0.545,0.66)	0.600	0.63 (0.564,0.682)	0.620	0.613 (0.545,0.661)	0.603	0.613 (0.545,0.661)	0.603	0.613 (0.545,0.661)	0.603
25	5	Equal	0.613 (0.551,0.66)	0.596	0.636 (0.558,0.695)	0.627	0.612 (0.545,0.662)	0.597	0.612 (0.545,0.662)	0.597	0.612 (0.545,0.662)	0.597
25	10	Equal	0.601 (0.54,0.643)	0.584	0.641 (0.565,0.705)	0.635	0.604 (0.542,0.647)	0.590	0.604 (0.542,0.647)	0.590	0.602 (0.543,0.643)	0.590
25	15	Equal	0.606 (0.544,0.654)	0.590	0.649 (0.571,0.712)	0.643	0.606 (0.545,0.649)	0.590	0.606 (0.545,0.649)	0.590	0.604 (0.545,0.649)	0.584
100	3	Equal	0.593 (0.55,0.631)	0.592	0.603 (0.563,0.64)	0.602	0.594 (0.549,0.632)	0.592	0.594 (0.549,0.632)	0.592	0.594 (0.549,0.632)	0.592
100	5	Equal	0.596 (0.555,0.633)	0.592	0.617 (0.581,0.654)	0.616	0.597 (0.555,0.633)	0.593	0.597 (0.555,0.633)	0.593	0.597 (0.555,0.633)	0.593
100	10	Equal	0.596 (0.553,0.632)	0.594	0.629 (0.591,0.667)	0.629	0.597 (0.554,0.633)	0.597	0.597 (0.554,0.633)	0.597	0.597 (0.554,0.633)	0.597
100	15	Equal	0.591 (0.55,0.628)	0.589	0.639 (0.602,0.678)	0.639	0.593 (0.552,0.629)	0.590	0.593 (0.552,0.629)	0.590	0.593 (0.552,0.629)	0.590
200	3	Equal	0.602 (0.574,0.629)	0.604	0.602 (0.574,0.63)	0.604	0.602 (0.576,0.629)	0.605	0.602 (0.576,0.629)	0.605	0.602 (0.576,0.629)	0.605
200	5	Equal	0.608 (0.58,0.637)	0.608	0.614 (0.586,0.644)	0.614	0.608 (0.58,0.638)	0.609	0.608 (0.58,0.638)	0.609	0.608 (0.58,0.638)	0.609
200	10	Equal	0.612 (0.583,0.642)	0.614	0.63 (0.604,0.657)	0.631	0.613 (0.584,0.643)	0.614	0.613 (0.584,0.643)	0.614	0.613 (0.584,0.643)	0.614
200	15	Equal	0.616 (0.586,0.646)	0.616	0.642 (0.615,0.669)	0.643	0.617 (0.587,0.646)	0.616	0.617 (0.587,0.646)	0.616	0.617 (0.587,0.646)	0.616
25	3	Not Equal	0.613 (0.545,0.662)	0.603	0.63 (0.564,0.682)	0.622	0.614 (0.545,0.667)	0.604	0.614 (0.545,0.667)	0.604	0.614 (0.545,0.667)	0.604
25	5	Not Equal	0.613 (0.551,0.663)	0.597	0.636 (0.558,0.695)	0.627	0.612 (0.545,0.662)	0.597	0.612 (0.545,0.662)	0.597	0.613 (0.545,0.662)	0.597
25	10	Not Equal	0.601 (0.54,0.647)	0.584	0.643 (0.567,0.707)	0.636	0.604 (0.545,0.647)	0.590	0.604 (0.545,0.647)	0.590	0.603 (0.545,0.647)	0.590
25	15	Not Equal	0.607 (0.539,0.654)	0.591	0.653 (0.577,0.718)	0.647	0.606 (0.54,0.649)	0.590	0.606 (0.54,0.649)	0.590	0.604 (0.54,0.65)	0.590
100	3	Not Equal	0.594 (0.551,0.632)	0.593	0.603 (0.563,0.641)	0.602	0.594 (0.552,0.633)	0.593	0.594 (0.552,0.633)	0.593	0.594 (0.552,0.633)	0.593
100	5	Not Equal	0.599 (0.558,0.636)	0.596	0.619 (0.581,0.656)	0.617	0.599 (0.557,0.637)	0.597	0.599 (0.557,0.637)	0.597	0.599 (0.557,0.637)	0.597
100	10	Not Equal	0.6 (0.557,0.638)	0.600	0.632 (0.593,0.669)	0.631	0.601 (0.558,0.639)	0.601	0.601 (0.558,0.639)	0.601	0.601 (0.558,0.639)	0.601
100	15	Not Equal	0.596 (0.556,0.633)	0.594	0.643 (0.607,0.681)	0.643	0.597 (0.557,0.635)	0.595	0.597 (0.557,0.635)	0.595	0.597 (0.557,0.635)	0.595
200	3	Not Equal	0.603 (0.576,0.63)	0.606	0.603 (0.575,0.631)	0.604	0.603 (0.577,0.63)	0.606	0.603 (0.577,0.63)	0.606	0.603 (0.577,0.63)	0.606
200	5	Not Equal	0.61 (0.583,0.639)	0.611	0.615 (0.587,0.645)	0.615	0.611 (0.583,0.64)	0.611	0.611 (0.583,0.64)	0.611	0.611 (0.583,0.64)	0.611
200	10	Not Equal	0.617 (0.587,0.646)	0.618	0.633 (0.606,0.66)	0.633	0.617 (0.588,0.647)	0.619	0.617 (0.588,0.647)	0.619	0.617 (0.588,0.647)	0.619
200	15	Not Equal	0.621 (0.591,0.651)	0.622	0.646 (0.619,0.672)	0.646	0.622 (0.592,0.652)	0.622	0.622 (0.592,0.652)	0.622	0.622 (0.592,0.652)	0.622

Note: Statistics presented in this table were derived from test datasets described in Section 4.

**Table 3:** Mean AUC values with 95% confidence intervals obtained for Beta distributions.

			LogRes		MaxMutInf		MaxEnt		MinRelEnt		MinCrEnt	
**n**	**d**	**Covariance**	**Mean (95%CI)**	**Median**	**Mean (95%CI)**	**Median**	**Mean (95%CI)**	**Median**	**Mean (95%CI)**	**Median**	**Mean (95%CI)**	**Median**
25	3	Equal	0.631 (0.558,0.688)	0.617	0.648 (0.571,0.714)	0.641	0.63 (0.558,0.692)	0.617	0.63 (0.558,0.692)	0.617	0.63 (0.558,0.692)	0.617
25	5	Equal	0.622 (0.552,0.679)	0.610	0.656 (0.578,0.723)	0.649	0.623 (0.552,0.68)	0.610	0.623 (0.552,0.68)	0.610	0.623 (0.552,0.68)	0.610
25	10	Equal	0.614 (0.549,0.667)	0.597	0.669 (0.59,0.737)	0.667	0.616 (0.551,0.673)	0.604	0.616 (0.551,0.673)	0.604	0.615 (0.545,0.667)	0.604
25	15	Equal	0.611 (0.546,0.662)	0.593	0.679 (0.604,0.75)	0.679	0.614 (0.545,0.667)	0.600	0.614 (0.545,0.667)	0.600	0.61 (0.545,0.667)	0.596
100	3	Equal	0.626 (0.583,0.665)	0.628	0.63 (0.592,0.668)	0.630	0.626 (0.583,0.666)	0.629	0.626 (0.583,0.666)	0.629	0.626 (0.583,0.666)	0.629
100	5	Equal	0.634 (0.595,0.673)	0.636	0.646 (0.609,0.685)	0.646	0.634 (0.595,0.674)	0.637	0.634 (0.595,0.674)	0.637	0.634 (0.595,0.674)	0.637
100	10	Equal	0.636 (0.599,0.673)	0.637	0.663 (0.627,0.7)	0.664	0.637 (0.599,0.674)	0.638	0.637 (0.599,0.674)	0.638	0.637 (0.599,0.674)	0.638
100	15	Equal	0.635 (0.595,0.678)	0.635	0.676 (0.64,0.714)	0.677	0.636 (0.595,0.678)	0.636	0.636 (0.595,0.678)	0.636	0.636 (0.595,0.678)	0.636
200	3	Equal	0.638 (0.611,0.665)	0.640	0.631 (0.604,0.661)	0.633	0.638 (0.612,0.665)	0.640	0.638 (0.612,0.665)	0.640	0.638 (0.612,0.665)	0.640
200	5	Equal	0.648 (0.621,0.676)	0.649	0.645 (0.617,0.675)	0.646	0.648 (0.621,0.677)	0.650	0.648 (0.621,0.677)	0.650	0.648 (0.621,0.677)	0.650
200	10	Equal	0.658 (0.634,0.684)	0.658	0.664 (0.638,0.691)	0.664	0.659 (0.633,0.684)	0.659	0.659 (0.633,0.684)	0.659	0.659 (0.633,0.684)	0.659
200	15	Equal	0.665 (0.638,0.693)	0.666	0.679 (0.655,0.705)	0.680	0.666 (0.638,0.694)	0.666	0.666 (0.638,0.694)	0.666	0.666 (0.638,0.694)	0.666
25	3	Not Equal	0.632 (0.563,0.692)	0.620	0.648 (0.571,0.714)	0.643	0.631 (0.564,0.691)	0.620	0.631 (0.564,0.691)	0.620	0.631 (0.564,0.691)	0.620
25	5	Not Equal	0.624 (0.551,0.68)	0.615	0.658 (0.578,0.725)	0.651	0.625 (0.552,0.682)	0.613	0.625 (0.552,0.682)	0.613	0.625 (0.552,0.682)	0.613
25	10	Not Equal	0.616 (0.551,0.667)	0.600	0.672 (0.593,0.74)	0.667	0.618 (0.551,0.673)	0.604	0.618 (0.551,0.673)	0.604	0.617 (0.551,0.669)	0.607
25	15	Not Equal	0.614 (0.547,0.667)	0.597	0.686 (0.61,0.76)	0.686	0.616 (0.545,0.669)	0.603	0.616 (0.545,0.669)	0.603	0.612 (0.545,0.667)	0.597
100	3	Not Equal	0.628 (0.585,0.667)	0.629	0.631 (0.593,0.669)	0.630	0.628 (0.585,0.669)	0.630	0.628 (0.585,0.669)	0.630	0.628 (0.585,0.669)	0.630
100	5	Not Equal	0.638 (0.599,0.677)	0.641	0.649 (0.612,0.688)	0.648	0.638 (0.599,0.678)	0.641	0.638 (0.599,0.678)	0.641	0.638 (0.599,0.678)	0.641
100	10	Not Equal	0.642 (0.605,0.681)	0.644	0.668 (0.631,0.705)	0.669	0.643 (0.606,0.682)	0.645	0.643 (0.606,0.682)	0.645	0.643 (0.606,0.682)	0.645
100	15	Not Equal	0.643 (0.605,0.687)	0.643	0.682 (0.647,0.72)	0.684	0.643 (0.604,0.687)	0.644	0.643 (0.604,0.687)	0.644	0.643 (0.604,0.687)	0.644
200	3	Not Equal	0.64 (0.613,0.667)	0.642	0.632 (0.605,0.662)	0.634	0.64 (0.613,0.667)	0.642	0.64 (0.613,0.667)	0.642	0.64 (0.613,0.667)	0.642
200	5	Not Equal	0.652 (0.624,0.68)	0.653	0.647 (0.618,0.676)	0.648	0.652 (0.624,0.68)	0.653	0.652 (0.624,0.68)	0.653	0.652 (0.624,0.68)	0.653
200	10	Not Equal	0.665 (0.64,0.692)	0.665	0.67 (0.643,0.695)	0.670	0.665 (0.64,0.692)	0.665	0.665 (0.64,0.692)	0.665	0.665 (0.64,0.692)	0.665
200	15	Not Equal	0.673 (0.647,0.701)	0.674	0.685 (0.66,0.711)	0.687	0.674 (0.648,0.702)	0.674	0.674 (0.648,0.702)	0.674	0.674 (0.648,0.702)	0.674

Note: Statistics presented in this table were derived from test datasets described in Section 4.

**Table 4:** AUC values for the first five predictors in each dataset.

Variable	Prognostic	Diagnostic
radius_mean	0.611	0.938
texture_mean	0.535	0.776
perimeter_mean	0.613	0.947
area_mean	0.618	0.938
smoothness_mean	0.532	0.722

**Table 5:** Coefficients obtained for prognostic dataset.

Prognostic	LogRes	MaxMutInf	MaxEnt	MinRelEnt	MinCrEnt
radius_mean	−0.609209	0.537218	−2.03368	−2.03368	−2.03368
texture_mean	−0.0518859	0.995454	−0.159585	−0.159585	−0.159585
perimeter_mean	4.83955 × $10^{-5}$	0.0269349	−0.0419467	−0.0419467	−0.0419467
area_mean	0.00667978	0.0693375	2.45539	2.45539	2.45539
smoothness_mean	2.93637	0.115625	0.0284566	0.0284566	0.0284566

**Table 6:** Coefficients obtained for the diagnostic dataset.

Diagnostic	LogRes	MaxMutInf	MaxEnt	MinRelEnt	MinCrEnt
radius_mean	−6.27525	0.538316	−1.98606	−1.98606	−17.6992
texture_mean	0.3641	0.997358	1.69841	1.69841	1.57074
perimeter_mean	0.607157	0.0275477	7.14262	7.14262	13.7528
area_mean	0.0417776	0.0702387	−0.053743	−0.053743	10.5983
smoothness_mean	118.462	0.115856	1.74935	1.74935	1.67574

**Table 7:** Predictive ability of calculated linear combinations for prognostic data.

Metric	LogRes	MaxMutInf	MaxEnt	MinRelEnt	MinCrEnt
AUC	0.626	0.610	0.618	0.618	0.618
AUPRC	0.388	0.332	0.340	0.340	0.340
MCC	0.194	0.165	0.0203	0.0203	0.0203

**Table 8:** Predictive ability of calculated linear combinations for diagnostic data.

Metric	LogRes	MaxMutInf	MaxEnt	MinRelEnt	MinCrEnt
AUC	0.984	0.951	0.953	0.953	0.940
AUPRC	0.973	0.935	0.936	0.936	0.921
MCC	0.853	0.577	0.603	0.603	0.293

## Data Availability

Winconsin datasets can be found at https://archive.ics.uci.edu/dataset/17/breast+cancer+wisconsin+diagnostic and https://archive.ics.uci.edu/dataset/16/breast+cancer+wisconsin+prognostic (accessed on 17 April 2025).

## Supplementary Materials

The following supporting information can be downloaded at: https://www.mdpi.com/article/10.3390/e27090985/s1, Table S1: Mean AUPRC values with 95% confidence intervals obtained for multivariate Normal distributions in equal allocation settings. Table S2: Mean AUPRC values with 95% confidence intervals obtained for Gamma distributions in equal allocation settings. Table S3: Mean AUPRC values with 95% confidence intervals obtained for Beta distributions in equal allocation settings. Table S4: Mean MCC values with 95% confidence intervals obtained for multivariate Normal distributions in equal allocation settings. Table S5: Mean MCC values with 95% confidence intervals obtained for Gamma distributions in equal allocation settings. Table S6: Mean MCC values with 95% confidence intervals obtained for Beta distributions in equal allocation settings. Table S7: Mean AUC values with 95% confidence intervals obtained for multivariate Normal distributions in unequal allocation settings. Table S8: Mean AUC values with 95% confidence intervals obtained for Gamma distributions in unequal allocation settings. Table S9: Mean AUC values with 95% confidence intervals obtained for Beta distributions in unequal allocation settings. Table S10: Mean AUPRC values with 95% confidence intervals obtained for multivariate Normal distributions in unequal allocation settings. Table S11: Mean AUPRC values with 95% confidence intervals obtained for Gamma distributions in unequal allocation settings. Table S12: Mean AUPRC values with 95% confidence intervals obtained for Beta distributions in unequal allocation settings. Table S13: Mean MCC values with 95% confidence intervals obtained for multivariate Normal distributions in unequal allocation settings. Table S14: Mean MCC values with 95% confidence intervals obtained for Gamma distributions in unequal allocation settings. Table S15: Mean MCC values with 95% confidence intervals obtained for Beta distributions in unequal allocation settings.

## Abbreviations

The following abbreviations are used in this manuscript:

ROC	Receiver Operating Characteristics
AUC	Area under the ROC curve
AUPRC	Area under the precision recall curve
MCC	Matthews Correlation Coefficient
MaxMutInf	Maximum Mutual Information
MaxEnt	Maximum Entropy
MinCrEnt	Minimum Cross Entropy
MinRelEnt	Minimum Relative Entropy
LogRes	Logistic Regression
