# Supplementary material for: Entropy Methods on Finding Optimal Linear Combinations with an Application to Biomarkers
Source: Entropy (Basel). 2025 Sep 21;27(9):985. doi: 10.3390/e27090985 (PMC12469204; doi:10.3390/e27090985)
Supplement: Supplementary file 1 [file entropy-27-00985-s001.zip › entropy-3815490-supplementary.pdf]

entropy

MDPI

Article

Entropy Methods on Finding Optimal Linear Combinations with an Application to Biomarkers

Mehmet Sinan İyisoy [1], Pınar Özdemir [2]

[1] Department of Medical Education and Informatics, Necmettin Erbakan University, Konya 42090, Turkey
[2] Department of Biostatistics, Hacettepe University, Ankara 06100, Turkey; pbc.ozdemir@gmail.com
* Correspondence: siyisoy@gmail.com

Abstract

Identifying an optimal linear combination of continuous variables is a key objective in various fields of research, such as medicine. This manuscript explores the use of information-theoretical approaches used to establish these linear combinations. *Coefficients obtained from logistic regression can be used to construct such a linear combination, and this approach has been commonly adopted in the literature for comparison purposes.* The main contribution of this work is to propose novel ways of determining these linear combination coefficients by optimizing information-theoretical objective functions. Biomarkers are usually continuous measurements utilized to diagnose if a patient has the underlying disease. Certain disease contexts may lack high diagnostic power biomarkers, making their optimal combination a critical area of interest. We apply the above-mentioned novel methods to the problem of a combination of biomarkers. We assess the performance of our proposed methods against combinations derived from logistic regression coefficients, by comparing area under the ROC curve (AUC) values *and other metrics* in a broad simulation and a real life data application.

Keywords: linear combination; binary outcome;biomarkers

MSC: 62B10, 94A15

check for updates

Received: 28 July 2025
Revised: 16 September 2025
Accepted: 17 September 2025
Published: 21 September 2025

Citation: İyisoy, M.Sinan; Özdemir, P. Entropy Methods on Finding Optimal Linear Combinations with an Application to Biomarkers. *Entropy* **2025**, *1*, 0. https://doi.org/

Copyright: © 2025 by the authors. Licensee MDPI, Basel, Switzerland. This article is an open access article distributed under the terms and conditions of the Creative Commons Attribution (CC BY) license (https://creativecommons.org/licenses/by/4.0/).

S1. Supplementary Tables

We present here the results we obtained for unequal allocation settings where $n_0 = 2n_1$ where n_0 is the number of samples in y_0 group. We also present results for metrics AUPRC and MCC for equal and unequal allocation settings.

Table S1. Mean AUPRC values with 95% confidence intervals obtained for multivariate Normal distributions in equal allocation settings.

			LogRes		MaxMutInf		MaxEnt		MinRelEnt		MinCrEnt	
**n**	**d**	**Covariance**	**Mean (95%CI)**	**Median**	**Mean (95%CI)**	**Median**	**Mean (95%CI)**	**Median**	**Mean (95%CI)**	**Median**	**Mean (95%CI)**	**Median**
25	3	Equal	0.567 (0.475,0.658)	0.565	0.573 (0.475,0.665)	0.571	0.569 (0.474,0.66)	0.564	0.569 (0.475,0.66)	0.565	0.568 (0.475,0.658)	0.564
25	5	Equal	0.548 (0.455,0.634)	0.542	0.572 (0.484,0.661)	0.568	0.552 (0.461,0.638)	0.544	0.554 (0.462,0.641)	0.547	0.554 (0.46,0.644)	0.547
25	10	Equal	0.531 (0.456,0.603)	0.531	0.571 (0.491,0.657)	0.566	0.533 (0.452,0.61)	0.533	0.533 (0.452,0.61)	0.533	0.531 (0.453,0.606)	0.529
25	15	Equal	0.522 (0.434,0.602)	0.518	0.572 (0.485,0.652)	0.575	0.528 (0.442,0.608)	0.525	0.528 (0.442,0.608)	0.525	0.519 (0.436,0.603)	0.510
100	3	Equal	0.591 (0.544,0.64)	0.590	0.568 (0.524,0.616)	0.570	0.591 (0.545,0.641)	0.591	0.591 (0.545,0.641)	0.591	0.591 (0.545,0.64)	0.591
100	5	Equal	0.587 (0.536,0.636)	0.585	0.574 (0.526,0.619)	0.575	0.588 (0.536,0.638)	0.588	0.588 (0.537,0.637)	0.587	0.587 (0.537,0.637)	0.586
100	10	Equal	0.598 (0.554,0.644)	0.597	0.587 (0.541,0.631)	0.588	0.599 (0.553,0.645)	0.599	0.599 (0.554,0.643)	0.598	0.599 (0.554,0.645)	0.597
100	15	Equal	0.599 (0.55,0.646)	0.601	0.595 (0.55,0.641)	0.591	0.6 (0.551,0.649)	0.602	0.6 (0.552,0.647)	0.601	0.599 (0.55,0.648)	0.601
200	3	Equal	0.599 (0.567,0.634)	0.598	0.57 (0.532,0.606)	0.569	0.599 (0.568,0.635)	0.598	0.599 (0.568,0.635)	0.598	0.6 (0.568,0.635)	0.598
200	5	Equal	0.606 (0.572,0.64)	0.605	0.58 (0.546,0.612)	0.580	0.606 (0.573,0.639)	0.605	0.606 (0.573,0.64)	0.605	0.606 (0.572,0.639)	0.605
200	10	Equal	0.623 (0.591,0.656)	0.625	0.597 (0.565,0.629)	0.596	0.623 (0.59,0.657)	0.625	0.624 (0.592,0.657)	0.626	0.623 (0.59,0.657)	0.624
200	15	Equal	0.629 (0.593,0.666)	0.629	0.601 (0.569,0.631)	0.601	0.629 (0.594,0.665)	0.630	0.629 (0.594,0.666)	0.629	0.629 (0.594,0.666)	0.630
25	3	Not Equal	0.567 (0.473,0.658)	0.563	0.577 (0.479,0.668)	0.577	0.568 (0.473,0.654)	0.565	0.569 (0.475,0.654)	0.565	0.568 (0.475,0.654)	0.564
25	5	Not Equal	0.549 (0.458,0.635)	0.542	0.58 (0.49,0.665)	0.579	0.554 (0.463,0.641)	0.548	0.554 (0.462,0.64)	0.545	0.553 (0.463,0.639)	0.549
25	10	Not Equal	0.531 (0.451,0.607)	0.528	0.583 (0.5,0.668)	0.582	0.534 (0.451,0.612)	0.534	0.534 (0.451,0.612)	0.534	0.532 (0.452,0.605)	0.528
25	15	Not Equal	0.521 (0.434,0.601)	0.519	0.586 (0.495,0.668)	0.591	0.528 (0.442,0.61)	0.523	0.528 (0.442,0.609)	0.523	0.519 (0.436,0.598)	0.514
100	3	Not Equal	0.591 (0.544,0.641)	0.590	0.574 (0.529,0.622)	0.574	0.592 (0.544,0.642)	0.590	0.592 (0.545,0.641)	0.590	0.592 (0.545,0.642)	0.590
100	5	Not Equal	0.59 (0.539,0.638)	0.589	0.583 (0.536,0.631)	0.584	0.59 (0.538,0.639)	0.589	0.59 (0.538,0.64)	0.590	0.59 (0.54,0.64)	0.590
100	10	Not Equal	0.602 (0.556,0.647)	0.601	0.604 (0.558,0.648)	0.606	0.602 (0.557,0.649)	0.603	0.603 (0.558,0.649)	0.603	0.602 (0.557,0.65)	0.603
100	15	Not Equal	0.602 (0.552,0.65)	0.605	0.616 (0.571,0.663)	0.616	0.603 (0.553,0.652)	0.605	0.603 (0.553,0.652)	0.604	0.603 (0.552,0.652)	0.605
200	3	Not Equal	0.6 (0.568,0.636)	0.598	0.576 (0.537,0.61)	0.575	0.6 (0.567,0.635)	0.599	0.6 (0.567,0.636)	0.599	0.6 (0.567,0.636)	0.599
200	5	Not Equal	0.61 (0.574,0.643)	0.609	0.591 (0.557,0.624)	0.590	0.61 (0.576,0.644)	0.610	0.61 (0.576,0.644)	0.610	0.61 (0.575,0.644)	0.610
200	10	Not Equal	0.628 (0.595,0.663)	0.630	0.616 (0.585,0.647)	0.615	0.629 (0.595,0.663)	0.631	0.628 (0.595,0.662)	0.630	0.629 (0.595,0.662)	0.631
200	15	Not Equal	0.634 (0.597,0.671)	0.635	0.625 (0.593,0.654)	0.626	0.635 (0.599,0.672)	0.635	0.635 (0.598,0.671)	0.636	0.635 (0.599,0.671)	0.635

Note: Statistics presented in this table were derived from test datasets described in Section 4.

Table S2. Mean AUPRC values with 95% confidence intervals obtained for Gamma distributions in equal allocation settings.

			LogRes		MaxMutInf		MaxEnt		MinRelEnt		MinCrEnt	
**n**	**d**	**Covariance**	**Mean (95%CI)**	**Median**	**Mean (95%CI)**	**Median**	**Mean (95%CI)**	**Median**	**Mean (95%CI)**	**Median**	**Mean (95%CI)**	**Median**
25	3	Equal	0.547 (0.464,0.628)	0.547	0.574 (0.494,0.654)	0.575	0.557 (0.472,0.638)	0.556	0.556 (0.47,0.638)	0.552	0.557 (0.47,0.641)	0.555
25	5	Equal	0.535 (0.452,0.615)	0.531	0.571 (0.49,0.65)	0.569	0.543 (0.458,0.623)	0.536	0.543 (0.458,0.623)	0.535	0.544 (0.458,0.625)	0.535
25	10	Equal	0.519 (0.447,0.589)	0.515	0.579 (0.508,0.649)	0.578	0.526 (0.456,0.603)	0.518	0.526 (0.456,0.602)	0.517	0.523 (0.451,0.599)	0.516
25	15	Equal	0.515 (0.435,0.585)	0.516	0.589 (0.514,0.671)	0.588	0.522 (0.44,0.601)	0.517	0.522 (0.44,0.601)	0.517	0.515 (0.44,0.591)	0.514
100	3	Equal	0.579 (0.535,0.624)	0.579	0.586 (0.537,0.63)	0.587	0.583 (0.536,0.63)	0.584	0.583 (0.536,0.629)	0.584	0.584 (0.537,0.63)	0.584
100	5	Equal	0.582 (0.537,0.628)	0.579	0.598 (0.553,0.638)	0.596	0.586 (0.542,0.633)	0.582	0.585 (0.542,0.632)	0.584	0.585 (0.542,0.63)	0.583
100	10	Equal	0.581 (0.538,0.627)	0.583	0.611 (0.567,0.655)	0.610	0.584 (0.54,0.63)	0.586	0.584 (0.54,0.63)	0.586	0.584 (0.54,0.632)	0.586
100	15	Equal	0.578 (0.536,0.623)	0.579	0.622 (0.578,0.666)	0.624	0.58 (0.537,0.623)	0.582	0.581 (0.538,0.623)	0.584	0.58 (0.537,0.624)	0.583
200	3	Equal	0.595 (0.565,0.628)	0.595	0.593 (0.561,0.624)	0.593	0.597 (0.567,0.632)	0.598	0.597 (0.567,0.63)	0.598	0.597 (0.567,0.631)	0.597
200	5	Equal	0.6 (0.567,0.634)	0.599	0.603 (0.571,0.637)	0.603	0.602 (0.568,0.636)	0.601	0.602 (0.569,0.636)	0.600	0.602 (0.568,0.636)	0.600
200	10	Equal	0.604 (0.569,0.637)	0.602	0.619 (0.586,0.651)	0.618	0.606 (0.571,0.641)	0.603	0.606 (0.571,0.641)	0.603	0.606 (0.571,0.641)	0.604
200	15	Equal	0.607 (0.572,0.641)	0.608	0.628 (0.596,0.663)	0.629	0.609 (0.574,0.642)	0.609	0.609 (0.574,0.641)	0.609	0.609 (0.573,0.642)	0.609
25	3	Not Equal	0.548 (0.465,0.625)	0.547	0.576 (0.495,0.659)	0.577	0.557 (0.473,0.638)	0.557	0.557 (0.473,0.639)	0.556	0.557 (0.473,0.638)	0.557
25	5	Not Equal	0.535 (0.452,0.617)	0.531	0.576 (0.496,0.657)	0.572	0.544 (0.458,0.625)	0.539	0.543 (0.459,0.625)	0.538	0.544 (0.458,0.625)	0.539
25	10	Not Equal	0.52 (0.448,0.59)	0.519	0.588 (0.516,0.659)	0.589	0.526 (0.453,0.6)	0.520	0.526 (0.453,0.6)	0.520	0.523 (0.448,0.597)	0.516
25	15	Not Equal	0.515 (0.439,0.588)	0.510	0.601 (0.525,0.682)	0.603	0.522 (0.44,0.597)	0.514	0.521 (0.44,0.597)	0.514	0.516 (0.44,0.592)	0.513
100	3	Not Equal	0.582 (0.538,0.627)	0.583	0.591 (0.541,0.635)	0.592	0.586 (0.54,0.633)	0.587	0.586 (0.539,0.634)	0.587	0.586 (0.539,0.634)	0.586
100	5	Not Equal	0.587 (0.542,0.634)	0.585	0.607 (0.564,0.647)	0.605	0.591 (0.545,0.639)	0.589	0.591 (0.546,0.64)	0.590	0.591 (0.546,0.638)	0.590
100	10	Not Equal	0.588 (0.544,0.635)	0.592	0.626 (0.583,0.67)	0.627	0.591 (0.544,0.637)	0.593	0.591 (0.544,0.637)	0.594	0.591 (0.544,0.637)	0.593
100	15	Not Equal	0.586 (0.543,0.63)	0.587	0.642 (0.6,0.686)	0.643	0.588 (0.545,0.631)	0.589	0.588 (0.545,0.631)	0.589	0.588 (0.545,0.631)	0.589
200	3	Not Equal	0.599 (0.569,0.632)	0.601	0.598 (0.566,0.63)	0.599	0.602 (0.572,0.635)	0.602	0.602 (0.571,0.635)	0.602	0.602 (0.571,0.635)	0.602
200	5	Not Equal	0.607 (0.574,0.643)	0.606	0.612 (0.579,0.646)	0.613	0.609 (0.577,0.644)	0.608	0.609 (0.576,0.644)	0.607	0.609 (0.576,0.644)	0.607
200	10	Not Equal	0.615 (0.58,0.652)	0.614	0.636 (0.606,0.668)	0.636	0.617 (0.581,0.653)	0.616	0.617 (0.581,0.653)	0.616	0.617 (0.581,0.653)	0.616
200	15	Not Equal	0.619 (0.584,0.653)	0.620	0.65 (0.62,0.682)	0.651	0.621 (0.585,0.654)	0.621	0.62 (0.585,0.654)	0.621	0.621 (0.585,0.654)	0.621

Note: Statistics presented in this table were derived from test datasets described in Section 4.

Table S3. Mean AUPRC values with 95% confidence intervals obtained for Beta distributions in equal allocation settings.

			LogRes		MaxMutInf		MaxEnt		MinRelEnt		MinCrEnt	
**n**	**d**	**Covariance**	**Mean (95%CI)**	**Median**	**Mean (95%CI)**	**Median**	**Mean (95%CI)**	**Median**	**Mean (95%CI)**	**Median**	**Mean (95%CI)**	**Median**
25	3	Equal	0.577 (0.485,0.663)	0.582	0.638 (0.552,0.73)	0.643	0.602 (0.5,0.702)	0.606	0.602 (0.501,0.7)	0.606	0.601 (0.499,0.7)	0.606
25	5	Equal	0.557 (0.478,0.639)	0.553	0.629 (0.545,0.719)	0.631	0.585 (0.489,0.678)	0.588	0.585 (0.49,0.678)	0.588	0.585 (0.489,0.679)	0.587
25	10	Equal	0.535 (0.456,0.612)	0.527	0.614 (0.538,0.691)	0.610	0.55 (0.474,0.63)	0.545	0.549 (0.474,0.628)	0.545	0.547 (0.468,0.629)	0.540
25	15	Equal	0.523 (0.443,0.603)	0.516	0.617 (0.543,0.697)	0.619	0.53 (0.451,0.607)	0.528	0.529 (0.451,0.607)	0.527	0.523 (0.444,0.599)	0.520
100	3	Equal	0.61 (0.566,0.656)	0.611	0.622 (0.573,0.669)	0.622	0.617 (0.571,0.666)	0.618	0.617 (0.571,0.666)	0.618	0.617 (0.571,0.666)	0.618
100	5	Equal	0.617 (0.572,0.661)	0.618	0.632 (0.589,0.673)	0.631	0.627 (0.579,0.674)	0.630	0.627 (0.579,0.674)	0.630	0.627 (0.579,0.674)	0.629
100	10	Equal	0.615 (0.57,0.661)	0.616	0.641 (0.596,0.684)	0.643	0.627 (0.58,0.674)	0.628	0.627 (0.58,0.672)	0.628	0.627 (0.58,0.673)	0.628
100	15	Equal	0.615 (0.569,0.662)	0.616	0.653 (0.609,0.697)	0.655	0.625 (0.575,0.673)	0.629	0.625 (0.575,0.673)	0.629	0.625 (0.576,0.673)	0.629
200	3	Equal	0.626 (0.594,0.659)	0.627	0.624 (0.591,0.658)	0.624	0.63 (0.599,0.662)	0.630	0.63 (0.599,0.662)	0.630	0.63 (0.599,0.662)	0.630
200	5	Equal	0.634 (0.602,0.667)	0.633	0.632 (0.598,0.668)	0.632	0.639 (0.607,0.674)	0.638	0.639 (0.607,0.674)	0.638	0.639 (0.607,0.674)	0.638
200	10	Equal	0.643 (0.608,0.678)	0.643	0.649 (0.617,0.682)	0.648	0.65 (0.616,0.684)	0.649	0.65 (0.616,0.684)	0.649	0.65 (0.616,0.684)	0.649
200	15	Equal	0.648 (0.616,0.683)	0.648	0.66 (0.629,0.694)	0.661	0.655 (0.622,0.689)	0.655	0.655 (0.622,0.689)	0.655	0.655 (0.622,0.689)	0.655
25	3	Not Equal	0.578 (0.488,0.667)	0.581	0.644 (0.56,0.732)	0.650	0.604 (0.502,0.703)	0.608	0.603 (0.502,0.701)	0.608	0.604 (0.503,0.703)	0.609
25	5	Not Equal	0.559 (0.475,0.641)	0.558	0.636 (0.556,0.719)	0.640	0.589 (0.493,0.682)	0.588	0.588 (0.492,0.684)	0.588	0.589 (0.496,0.684)	0.589
25	10	Not Equal	0.539 (0.462,0.617)	0.534	0.631 (0.559,0.709)	0.632	0.554 (0.475,0.63)	0.551	0.554 (0.475,0.632)	0.552	0.551 (0.468,0.63)	0.547
25	15	Not Equal	0.525 (0.44,0.608)	0.519	0.634 (0.559,0.715)	0.642	0.532 (0.451,0.614)	0.528	0.532 (0.452,0.614)	0.528	0.525 (0.441,0.602)	0.522
100	3	Not Equal	0.616 (0.573,0.663)	0.616	0.629 (0.581,0.675)	0.630	0.624 (0.578,0.671)	0.626	0.624 (0.578,0.671)	0.626	0.624 (0.578,0.671)	0.626
100	5	Not Equal	0.625 (0.579,0.67)	0.627	0.644 (0.6,0.683)	0.643	0.636 (0.589,0.683)	0.640	0.636 (0.589,0.683)	0.640	0.636 (0.589,0.683)	0.640
100	10	Not Equal	0.627 (0.584,0.674)	0.628	0.66 (0.615,0.703)	0.663	0.64 (0.595,0.687)	0.643	0.639 (0.595,0.685)	0.643	0.639 (0.595,0.687)	0.643
100	15	Not Equal	0.627 (0.582,0.675)	0.629	0.677 (0.636,0.72)	0.679	0.638 (0.59,0.687)	0.643	0.638 (0.591,0.687)	0.642	0.638 (0.591,0.685)	0.642
200	3	Not Equal	0.634 (0.603,0.667)	0.634	0.632 (0.599,0.667)	0.632	0.638 (0.607,0.671)	0.639	0.638 (0.607,0.671)	0.639	0.638 (0.607,0.671)	0.639
200	5	Not Equal	0.645 (0.614,0.678)	0.646	0.645 (0.613,0.68)	0.648	0.651 (0.62,0.686)	0.651	0.651 (0.62,0.686)	0.651	0.651 (0.62,0.686)	0.651
200	10	Not Equal	0.66 (0.626,0.695)	0.661	0.67 (0.639,0.701)	0.671	0.667 (0.634,0.702)	0.668	0.667 (0.634,0.702)	0.669	0.667 (0.634,0.702)	0.669
200	15	Not Equal	0.666 (0.634,0.701)	0.666	0.686 (0.656,0.718)	0.688	0.673 (0.64,0.707)	0.674	0.673 (0.64,0.707)	0.674	0.673 (0.64,0.708)	0.674

Note: Statistics presented in this table were derived from test datasets described in Section 4.

Table S4. Mean MCC values with 95% confidence intervals obtained for multivariate Normal distributions in equal allocation settings

			**LogRes**		**MaxMutInf**		**MaxEnt**		**MinRelEnt**		**MinCrEnt**	
**n**	**d**	**Covariance**	**Mean (95%CI)**	**Median**	**Mean (95%CI)**	**Median**	**Mean (95%CI)**	**Median**	**Mean (95%CI)**	**Median**	**Mean (95%CI)**	**Median**
25	3	Equal	0.073 (-0.068,0.212)	0.068	0.098 (-0.038,0.24)	0.116	0.077 (-0.068,0.22)	0.082	0.077 (-0.068,0.22)	0.082	0.077 (-0.068,0.22)	0.082
25	5	Equal	0.074 (-0.068,0.22)	0.089	0.121 (-0.015,0.263)	0.139	0.074 (-0.079,0.22)	0.080	0.074 (-0.079,0.22)	0.080	0.074 (-0.079,0.22)	0.080
25	10	Equal	0.058 (-0.08,0.21)	0.067	0.144 (0.017,0.28)	0.167	0.062 (-0.068,0.211)	0.076	0.062 (-0.068,0.211)	0.076	0.058 (-0.083,0.21)	0.068
25	15	Equal	0.059 (-0.113,0.212)	0.068	0.145 (0.012,0.28)	0.165	0.062 (-0.099,0.214)	0.068	0.062 (-0.099,0.214)	0.068	0.052 (-0.09,0.201)	0.053
100	3	Equal	0.111 (0.042,0.18)	0.116	0.094 (0.027,0.163)	0.095	0.115 (0.045,0.181)	0.119	0.115 (0.045,0.181)	0.119	0.115 (0.045,0.181)	0.119
100	5	Equal	0.112 (0.042,0.187)	0.118	0.11 (0.043,0.178)	0.106	0.119 (0.05,0.192)	0.119	0.119 (0.05,0.192)	0.119	0.119 (0.05,0.192)	0.119
100	10	Equal	0.131 (0.061,0.207)	0.134	0.125 (0.059,0.189)	0.123	0.134 (0.068,0.203)	0.134	0.134 (0.068,0.203)	0.134	0.134 (0.068,0.203)	0.134
100	15	Equal	0.138 (0.07,0.204)	0.142	0.141 (0.076,0.211)	0.144	0.139 (0.069,0.213)	0.139	0.139 (0.069,0.213)	0.139	0.139 (0.069,0.213)	0.139
200	3	Equal	0.128 (0.078,0.18)	0.131	0.094 (0.044,0.149)	0.093	0.125 (0.077,0.175)	0.125	0.125 (0.077,0.175)	0.125	0.125 (0.077,0.175)	0.125
200	5	Equal	0.137 (0.091,0.185)	0.138	0.111 (0.061,0.164)	0.111	0.138 (0.091,0.188)	0.139	0.138 (0.091,0.188)	0.139	0.138 (0.091,0.188)	0.139
200	10	Equal	0.161 (0.105,0.217)	0.163	0.132 (0.08,0.183)	0.131	0.159 (0.106,0.216)	0.160	0.159 (0.106,0.216)	0.160	0.159 (0.106,0.216)	0.160
200	15	Equal	0.17 (0.116,0.225)	0.172	0.138 (0.087,0.188)	0.136	0.171 (0.114,0.226)	0.172	0.171 (0.114,0.226)	0.172	0.171 (0.114,0.226)	0.172
25	3	Not Equal	0.071 (-0.079,0.212)	0.068	0.098 (-0.042,0.238)	0.120	0.078 (-0.068,0.22)	0.083	0.078 (-0.068,0.22)	0.083	0.078 (-0.068,0.22)	0.083
25	5	Not Equal	0.077 (-0.068,0.23)	0.090	0.122 (-0.017,0.257)	0.132	0.078 (-0.066,0.23)	0.080	0.078 (-0.066,0.23)	0.080	0.078 (-0.066,0.23)	0.080
25	10	Not Equal	0.068 (-0.079,0.21)	0.079	0.146 (0.014,0.28)	0.167	0.074 (-0.066,0.21)	0.094	0.074 (-0.066,0.21)	0.094	0.069 (-0.079,0.206)	0.083
25	15	Not Equal	0.06 (-0.1,0.212)	0.068	0.153 (0.012,0.292)	0.175	0.074 (-0.083,0.23)	0.089	0.074 (-0.083,0.23)	0.089	0.06 (-0.089,0.21)	0.066
100	3	Not Equal	0.118 (0.052,0.186)	0.118	0.096 (0.024,0.168)	0.100	0.116 (0.05,0.186)	0.116	0.116 (0.05,0.186)	0.116	0.116 (0.05,0.186)	0.116
100	5	Not Equal	0.117 (0.047,0.191)	0.117	0.112 (0.04,0.18)	0.115	0.121 (0.048,0.194)	0.120	0.121 (0.048,0.194)	0.120	0.121 (0.048,0.194)	0.120
100	10	Not Equal	0.133 (0.064,0.204)	0.137	0.134 (0.065,0.206)	0.139	0.138 (0.069,0.21)	0.140	0.138 (0.069,0.21)	0.140	0.138 (0.069,0.21)	0.140
100	15	Not Equal	0.138 (0.077,0.207)	0.139	0.146 (0.078,0.215)	0.148	0.146 (0.077,0.217)	0.146	0.146 (0.077,0.217)	0.146	0.146 (0.077,0.217)	0.146
200	3	Not Equal	0.127 (0.077,0.176)	0.127	0.097 (0.041,0.152)	0.099	0.127 (0.078,0.18)	0.128	0.127 (0.078,0.18)	0.128	0.127 (0.078,0.18)	0.128
200	5	Not Equal	0.136 (0.086,0.188)	0.137	0.115 (0.063,0.167)	0.112	0.141 (0.089,0.19)	0.139	0.141 (0.089,0.19)	0.139	0.141 (0.089,0.19)	0.139
200	10	Not Equal	0.165 (0.113,0.219)	0.170	0.137 (0.087,0.191)	0.134	0.163 (0.113,0.219)	0.163	0.163 (0.113,0.219)	0.163	0.163 (0.113,0.219)	0.163
200	15	Not Equal	0.173 (0.12,0.227)	0.174	0.147 (0.098,0.201)	0.150	0.177 (0.124,0.231)	0.179	0.177 (0.124,0.231)	0.179	0.177 (0.124,0.231)	0.179

Note: Statistics presented in this table were derived from test datasets described in Section 4.

Table S5. Mean MCC values with 95% confidence intervals obtained for Gamma distributions in equal allocation settings

			LogRes		MaxMutInf		MaxEnt		MinRelEnt		MinCrEnt	
**n**	**d**	**Covariance**	**Mean (95%CI)**	**Median**	**Mean (95%CI)**	**Median**	**Mean (95%CI)**	**Median**	**Mean (95%CI)**	**Median**	**Mean (95%CI)**	**Median**
25	3	Equal	0.069 (-0.08,0.221)	0.072	0.132 (-0.007,0.263)	0.161	0.075 (-0.066,0.221)	0.079	0.075 (-0.066,0.221)	0.079	0.075 (-0.066,0.221)	0.079
25	5	Equal	0.057 (-0.098,0.204)	0.064	0.136 (0,0.282)	0.161	0.059 (-0.108,0.208)	0.064	0.059 (-0.108,0.208)	0.064	0.06 (-0.102,0.206)	0.065
25	10	Equal	0.048 (-0.108,0.196)	0.053	0.152 (0.022,0.283)	0.169	0.053 (-0.1,0.206)	0.053	0.053 (-0.1,0.206)	0.053	0.05 (-0.102,0.199)	0.045
25	15	Equal	0.037 (-0.116,0.196)	0.045	0.162 (0.04,0.283)	0.181	0.036 (-0.114,0.196)	0.033	0.036 (-0.114,0.196)	0.033	0.024 (-0.138,0.194)	0.031
100	3	Equal	0.106 (0.042,0.178)	0.109	0.122 (0.058,0.194)	0.123	0.107 (0.038,0.178)	0.110	0.107 (0.038,0.178)	0.110	0.107 (0.038,0.178)	0.110
100	5	Equal	0.11 (0.034,0.19)	0.112	0.142 (0.078,0.207)	0.142	0.107 (0.033,0.182)	0.107	0.107 (0.033,0.182)	0.107	0.107 (0.033,0.182)	0.107
100	10	Equal	0.107 (0.038,0.181)	0.111	0.148 (0.079,0.22)	0.149	0.108 (0.038,0.177)	0.112	0.108 (0.038,0.177)	0.112	0.108 (0.038,0.177)	0.112
100	15	Equal	0.102 (0.032,0.174)	0.107	0.165 (0.101,0.231)	0.167	0.106 (0.04,0.177)	0.111	0.106 (0.04,0.177)	0.111	0.106 (0.04,0.177)	0.111
200	3	Equal	0.126 (0.078,0.175)	0.131	0.127 (0.077,0.176)	0.127	0.122 (0.071,0.172)	0.122	0.122 (0.071,0.172)	0.122	0.122 (0.071,0.172)	0.122
200	5	Equal	0.129 (0.078,0.181)	0.129	0.136 (0.085,0.184)	0.137	0.132 (0.082,0.183)	0.131	0.132 (0.082,0.183)	0.131	0.132 (0.082,0.183)	0.131
200	10	Equal	0.133 (0.081,0.189)	0.137	0.159 (0.109,0.212)	0.162	0.137 (0.087,0.188)	0.139	0.137 (0.087,0.188)	0.139	0.137 (0.087,0.188)	0.139
200	15	Equal	0.143 (0.095,0.194)	0.140	0.166 (0.118,0.22)	0.170	0.142 (0.09,0.194)	0.141	0.142 (0.09,0.194)	0.141	0.142 (0.09,0.194)	0.141
25	3	Not Equal	0.066 (-0.068,0.21)	0.068	0.129 (-0.014,0.263)	0.161	0.07 (-0.066,0.22)	0.066	0.07 (-0.066,0.22)	0.066	0.07 (-0.066,0.22)	0.066
25	5	Not Equal	0.059 (-0.105,0.211)	0.064	0.139 (0.007,0.282)	0.165	0.061 (-0.102,0.212)	0.068	0.061 (-0.102,0.212)	0.068	0.062 (-0.1,0.212)	0.068
25	10	Not Equal	0.044 (-0.102,0.199)	0.038	0.159 (0.035,0.294)	0.181	0.051 (-0.1,0.201)	0.064	0.051 (-0.1,0.201)	0.064	0.048 (-0.102,0.2)	0.053
25	15	Not Equal	0.032 (-0.13,0.197)	0.040	0.165 (0.046,0.283)	0.196	0.035 (-0.116,0.196)	0.027	0.035 (-0.116,0.196)	0.027	0.034 (-0.136,0.196)	0.038
100	3	Not Equal	0.108 (0.036,0.179)	0.114	0.12 (0.055,0.184)	0.120	0.11 (0.037,0.18)	0.111	0.11 (0.037,0.18)	0.111	0.11 (0.037,0.18)	0.111
100	5	Not Equal	0.117 (0.048,0.192)	0.123	0.147 (0.087,0.21)	0.149	0.114 (0.045,0.188)	0.117	0.114 (0.045,0.188)	0.117	0.114 (0.045,0.188)	0.117
100	10	Not Equal	0.114 (0.049,0.182)	0.117	0.156 (0.089,0.225)	0.160	0.116 (0.043,0.186)	0.122	0.116 (0.043,0.186)	0.122	0.116 (0.043,0.186)	0.122
100	15	Not Equal	0.109 (0.041,0.178)	0.113	0.174 (0.103,0.248)	0.175	0.11 (0.045,0.179)	0.116	0.11 (0.045,0.179)	0.116	0.11 (0.045,0.179)	0.116
200	3	Not Equal	0.124 (0.075,0.176)	0.126	0.124 (0.073,0.18)	0.130	0.124 (0.074,0.175)	0.126	0.124 (0.074,0.175)	0.126	0.124 (0.074,0.175)	0.126
200	5	Not Equal	0.132 (0.08,0.183)	0.133	0.137 (0.08,0.193)	0.142	0.134 (0.088,0.184)	0.136	0.134 (0.088,0.184)	0.136	0.134 (0.088,0.184)	0.136
200	10	Not Equal	0.142 (0.088,0.197)	0.144	0.162 (0.108,0.219)	0.166	0.143 (0.095,0.196)	0.146	0.143 (0.095,0.196)	0.146	0.143 (0.095,0.196)	0.146
200	15	Not Equal	0.147 (0.095,0.2)	0.148	0.171 (0.118,0.231)	0.176	0.145 (0.092,0.2)	0.146	0.145 (0.092,0.2)	0.146	0.145 (0.092,0.2)	0.146

Note: Statistics presented in this table were derived from test datasets described in Section 4.

Table S6. Mean MCC values with 95% confidence intervals obtained for Beta distributions in equal allocation settings

			LogRes		MaxMutInf		MaxEnt		MinRelEnt		MinCrEnt	
**n**	**d**	**Covariance**	**Mean (95%CI)**	**Median**	**Mean (95%CI)**	**Median**	**Mean (95%CI)**	**Median**	**Mean (95%CI)**	**Median**	**Mean (95%CI)**	**Median**
25	3	Equal	0.106 (-0.04,0.25)	0.116	0.162 (0.04,0.292)	0.181	0.105 (-0.045,0.257)	0.122	0.105 (-0.045,0.257)	0.122	0.105 (-0.045,0.257)	0.122
25	5	Equal	0.093 (-0.053,0.241)	0.114	0.178 (0.042,0.317)	0.196	0.099 (-0.04,0.243)	0.116	0.099 (-0.04,0.243)	0.116	0.099 (-0.045,0.243)	0.116
25	10	Equal	0.082 (-0.064,0.225)	0.090	0.194 (0.068,0.327)	0.206	0.077 (-0.06,0.212)	0.080	0.077 (-0.06,0.212)	0.080	0.074 (-0.066,0.212)	0.086
25	15	Equal	0.065 (-0.097,0.212)	0.066	0.211 (0.101,0.345)	0.220	0.072 (-0.068,0.22)	0.089	0.072 (-0.068,0.22)	0.089	0.052 (-0.099,0.206)	0.053
100	3	Equal	0.149 (0.083,0.224)	0.153	0.157 (0.093,0.221)	0.154	0.154 (0.082,0.227)	0.157	0.154 (0.082,0.227)	0.157	0.154 (0.082,0.227)	0.157
100	5	Equal	0.156 (0.09,0.225)	0.156	0.176 (0.105,0.245)	0.176	0.154 (0.09,0.223)	0.156	0.154 (0.09,0.223)	0.156	0.154 (0.09,0.223)	0.156
100	10	Equal	0.158 (0.093,0.23)	0.162	0.195 (0.132,0.264)	0.196	0.158 (0.092,0.233)	0.161	0.158 (0.092,0.233)	0.161	0.158 (0.092,0.233)	0.161
100	15	Equal	0.157 (0.087,0.225)	0.162	0.208 (0.139,0.28)	0.209	0.157 (0.09,0.224)	0.160	0.157 (0.09,0.224)	0.160	0.157 (0.09,0.224)	0.160
200	3	Equal	0.17 (0.12,0.224)	0.169	0.159 (0.107,0.211)	0.160	0.169 (0.119,0.225)	0.171	0.169 (0.119,0.225)	0.171	0.169 (0.119,0.225)	0.171
200	5	Equal	0.181 (0.131,0.233)	0.183	0.173 (0.12,0.233)	0.173	0.18 (0.128,0.231)	0.180	0.18 (0.128,0.231)	0.180	0.18 (0.128,0.231)	0.180
200	10	Equal	0.189 (0.136,0.245)	0.193	0.197 (0.151,0.25)	0.196	0.191 (0.139,0.243)	0.192	0.191 (0.139,0.243)	0.192	0.191 (0.139,0.243)	0.192
200	15	Equal	0.196 (0.142,0.253)	0.197	0.216 (0.167,0.271)	0.221	0.2 (0.148,0.254)	0.201	0.2 (0.148,0.254)	0.201	0.2 (0.148,0.254)	0.201
25	3	Not Equal	0.113 (-0.036,0.263)	0.131	0.164 (0.053,0.299)	0.188	0.113 (-0.036,0.263)	0.131	0.113 (-0.036,0.263)	0.131	0.113 (-0.036,0.263)	0.131
25	5	Not Equal	0.102 (-0.045,0.256)	0.120	0.176 (0.036,0.316)	0.196	0.106 (-0.043,0.263)	0.131	0.106 (-0.043,0.263)	0.131	0.105 (-0.048,0.263)	0.122
25	10	Not Equal	0.087 (-0.051,0.241)	0.099	0.208 (0.085,0.351)	0.218	0.089 (-0.053,0.238)	0.108	0.089 (-0.053,0.238)	0.108	0.086 (-0.053,0.23)	0.108
25	15	Not Equal	0.064 (-0.089,0.212)	0.060	0.216 (0.09,0.356)	0.221	0.078 (-0.068,0.23)	0.086	0.078 (-0.068,0.23)	0.086	0.05 (-0.12,0.212)	0.053
100	3	Not Equal	0.155 (0.091,0.229)	0.159	0.155 (0.089,0.222)	0.157	0.155 (0.085,0.231)	0.155	0.155 (0.085,0.231)	0.155	0.155 (0.085,0.231)	0.155
100	5	Not Equal	0.162 (0.099,0.231)	0.165	0.183 (0.116,0.246)	0.181	0.162 (0.097,0.23)	0.169	0.162 (0.097,0.23)	0.169	0.162 (0.097,0.23)	0.169
100	10	Not Equal	0.174 (0.104,0.25)	0.176	0.202 (0.132,0.273)	0.203	0.173 (0.101,0.247)	0.176	0.173 (0.101,0.247)	0.176	0.173 (0.101,0.247)	0.176
100	15	Not Equal	0.173 (0.105,0.246)	0.176	0.217 (0.146,0.293)	0.220	0.17 (0.096,0.247)	0.173	0.17 (0.096,0.247)	0.173	0.17 (0.096,0.247)	0.173
200	3	Not Equal	0.169 (0.117,0.221)	0.172	0.16 (0.103,0.216)	0.164	0.17 (0.116,0.221)	0.172	0.17 (0.116,0.221)	0.172	0.17 (0.116,0.221)	0.172
200	5	Not Equal	0.181 (0.127,0.237)	0.182	0.173 (0.119,0.227)	0.173	0.182 (0.126,0.238)	0.183	0.182 (0.126,0.238)	0.183	0.182 (0.126,0.238)	0.183
200	10	Not Equal	0.197 (0.142,0.252)	0.197	0.203 (0.151,0.26)	0.207	0.193 (0.138,0.25)	0.196	0.193 (0.138,0.25)	0.196	0.193 (0.138,0.25)	0.196
200	15	Not Equal	0.207 (0.145,0.265)	0.209	0.223 (0.167,0.288)	0.228	0.209 (0.156,0.263)	0.216	0.209 (0.156,0.263)	0.216	0.209 (0.156,0.263)	0.216

Note: Statistics presented in this table were derived from test datasets described in Section 4.

Table S7. Mean AUC values with 95% confidence intervals obtained for multivariate Normal distributions in unequal allocation settings

			LogRes		MaxMutInf		MaxEnt		MinRelEnt		MinCrEnt	
**n**	**d**	**Covariance**	**Mean (95%CI)**	**Median**	**Mean (95%CI)**	**Median**	**Mean (95%CI)**	**Median**	**Mean (95%CI)**	**Median**	**Mean (95%CI)**	**Median**
25	3	Equal	0.644 (0.558,0.711)	0.625	0.643 (0.558,0.708)	0.625	0.644 (0.558,0.708)	0.625	0.644 (0.558,0.708)	0.625	0.644 (0.558,0.708)	0.625
25	5	Equal	0.642 (0.558,0.711)	0.623	0.639 (0.558,0.701)	0.623	0.641 (0.556,0.708)	0.623	0.641 (0.556,0.708)	0.623	0.641 (0.556,0.708)	0.623
25	10	Equal	0.628 (0.554,0.688)	0.613	0.646 (0.558,0.714)	0.625	0.628 (0.554,0.688)	0.613	0.628 (0.554,0.688)	0.613	0.625 (0.554,0.681)	0.607
25	15	Equal	0.634 (0.556,0.693)	0.622	0.66 (0.569,0.738)	0.643	0.629 (0.554,0.692)	0.611	0.629 (0.554,0.692)	0.611	0.631 (0.556,0.692)	0.612
100	3	Equal	0.602 (0.552,0.646)	0.600	0.593 (0.542,0.633)	0.585	0.603 (0.552,0.647)	0.599	0.603 (0.552,0.647)	0.599	0.603 (0.552,0.647)	0.599
100	5	Equal	0.598 (0.548,0.64)	0.592	0.596 (0.548,0.635)	0.590	0.598 (0.549,0.64)	0.593	0.598 (0.549,0.64)	0.593	0.598 (0.549,0.64)	0.593
100	10	Equal	0.605 (0.553,0.648)	0.602	0.616 (0.568,0.66)	0.615	0.606 (0.554,0.649)	0.602	0.606 (0.554,0.649)	0.602	0.606 (0.554,0.649)	0.602
100	15	Equal	0.606 (0.554,0.65)	0.602	0.622 (0.572,0.665)	0.620	0.608 (0.555,0.65)	0.604	0.608 (0.555,0.65)	0.604	0.608 (0.555,0.65)	0.604
200	3	Equal	0.604 (0.569,0.638)	0.605	0.585 (0.548,0.618)	0.581	0.605 (0.568,0.638)	0.606	0.605 (0.568,0.638)	0.606	0.605 (0.568,0.638)	0.606
200	5	Equal	0.608 (0.573,0.641)	0.607	0.593 (0.556,0.629)	0.593	0.608 (0.574,0.641)	0.608	0.608 (0.574,0.641)	0.608	0.608 (0.574,0.641)	0.608
200	10	Equal	0.623 (0.588,0.659)	0.622	0.611 (0.578,0.644)	0.609	0.624 (0.588,0.659)	0.623	0.624 (0.588,0.659)	0.623	0.624 (0.588,0.659)	0.623
200	15	Equal	0.628 (0.594,0.663)	0.629	0.615 (0.583,0.648)	0.616	0.629 (0.593,0.664)	0.631	0.629 (0.593,0.664)	0.631	0.629 (0.593,0.664)	0.631
25	3	Not Equal	0.644 (0.558,0.709)	0.625	0.645 (0.561,0.709)	0.625	0.645 (0.562,0.713)	0.625	0.645 (0.562,0.713)	0.625	0.645 (0.562,0.713)	0.625
25	5	Not Equal	0.642 (0.556,0.714)	0.625	0.641 (0.556,0.701)	0.625	0.641 (0.556,0.708)	0.623	0.641 (0.556,0.708)	0.623	0.641 (0.556,0.708)	0.623
25	10	Not Equal	0.629 (0.556,0.681)	0.611	0.649 (0.558,0.722)	0.631	0.627 (0.554,0.684)	0.613	0.627 (0.554,0.684)	0.613	0.625 (0.554,0.688)	0.611
25	15	Not Equal	0.634 (0.556,0.692)	0.615	0.666 (0.569,0.743)	0.649	0.629 (0.554,0.688)	0.613	0.629 (0.554,0.688)	0.613	0.633 (0.556,0.692)	0.611
100	3	Not Equal	0.603 (0.552,0.646)	0.600	0.594 (0.542,0.633)	0.586	0.603 (0.552,0.647)	0.600	0.603 (0.552,0.647)	0.600	0.603 (0.552,0.647)	0.600
100	5	Not Equal	0.6 (0.549,0.642)	0.593	0.598 (0.55,0.641)	0.592	0.6 (0.549,0.642)	0.594	0.6 (0.549,0.642)	0.594	0.6 (0.549,0.642)	0.594
100	10	Not Equal	0.608 (0.557,0.654)	0.605	0.62 (0.571,0.664)	0.620	0.61 (0.558,0.655)	0.606	0.61 (0.558,0.655)	0.606	0.61 (0.558,0.655)	0.606
100	15	Not Equal	0.609 (0.559,0.653)	0.604	0.627 (0.579,0.674)	0.626	0.612 (0.562,0.656)	0.608	0.612 (0.562,0.656)	0.608	0.612 (0.562,0.656)	0.608
200	3	Not Equal	0.605 (0.568,0.639)	0.606	0.586 (0.55,0.62)	0.581	0.605 (0.568,0.638)	0.607	0.605 (0.568,0.638)	0.607	0.605 (0.568,0.638)	0.607
200	5	Not Equal	0.61 (0.576,0.643)	0.609	0.596 (0.559,0.632)	0.595	0.61 (0.575,0.644)	0.610	0.61 (0.575,0.644)	0.610	0.61 (0.575,0.644)	0.610
200	10	Not Equal	0.627 (0.591,0.662)	0.626	0.615 (0.581,0.65)	0.614	0.628 (0.592,0.663)	0.626	0.628 (0.592,0.663)	0.626	0.628 (0.592,0.663)	0.626
200	15	Not Equal	0.632 (0.598,0.667)	0.634	0.621 (0.588,0.655)	0.623	0.633 (0.599,0.669)	0.635	0.633 (0.599,0.669)	0.635	0.633 (0.599,0.669)	0.635

Note: Statistics presented in this table were derived from test datasets described in Section 4.

Table S8. Mean AUC values with 95% confidence intervals obtained for Gamma distributions in unequal allocation settings

			LogRes		MaxMutInf		MaxEnt		MinRelEnt		MinCrEnt	
**n**	**d**	**Covariance**	**Mean (95%CI)**	**Median**	**Mean (95%CI)**	**Median**	**Mean (95%CI)**	**Median**	**Mean (95%CI)**	**Median**	**Mean (95%CI)**	**Median**
25	3	Equal	0.631 (0.554,0.692)	0.612	0.647 (0.558,0.714)	0.631	0.631 (0.554,0.692)	0.615	0.631 (0.554,0.692)	0.615	0.631 (0.554,0.692)	0.615
25	5	Equal	0.634 (0.556,0.696)	0.613	0.653 (0.569,0.724)	0.636	0.633 (0.556,0.696)	0.615	0.633 (0.556,0.696)	0.615	0.633 (0.555,0.694)	0.615
25	10	Equal	0.63 (0.554,0.692)	0.611	0.664 (0.571,0.736)	0.649	0.627 (0.554,0.688)	0.610	0.627 (0.554,0.688)	0.610	0.629 (0.554,0.688)	0.611
25	15	Equal	0.629 (0.554,0.684)	0.611	0.667 (0.571,0.74)	0.653	0.628 (0.554,0.688)	0.611	0.628 (0.554,0.688)	0.611	0.628 (0.556,0.679)	0.610
100	3	Equal	0.594 (0.545,0.635)	0.587	0.608 (0.56,0.649)	0.604	0.595 (0.546,0.637)	0.587	0.595 (0.546,0.637)	0.587	0.595 (0.546,0.637)	0.587
100	5	Equal	0.593 (0.544,0.632)	0.586	0.619 (0.569,0.664)	0.612	0.594 (0.543,0.636)	0.587	0.594 (0.543,0.636)	0.587	0.594 (0.543,0.636)	0.587
100	10	Equal	0.589 (0.536,0.631)	0.582	0.633 (0.584,0.679)	0.631	0.591 (0.538,0.633)	0.585	0.591 (0.538,0.633)	0.585	0.591 (0.538,0.633)	0.585
100	15	Equal	0.585 (0.537,0.623)	0.575	0.639 (0.592,0.684)	0.641	0.588 (0.541,0.627)	0.580	0.588 (0.541,0.627)	0.580	0.588 (0.541,0.627)	0.580
200	3	Equal	0.595 (0.557,0.629)	0.594	0.602 (0.563,0.639)	0.601	0.595 (0.558,0.629)	0.594	0.595 (0.558,0.629)	0.594	0.595 (0.558,0.629)	0.594
200	5	Equal	0.598 (0.563,0.634)	0.599	0.611 (0.578,0.646)	0.611	0.599 (0.565,0.635)	0.599	0.599 (0.565,0.635)	0.599	0.599 (0.565,0.635)	0.599
200	10	Equal	0.599 (0.563,0.636)	0.596	0.63 (0.597,0.663)	0.633	0.601 (0.564,0.638)	0.598	0.601 (0.564,0.638)	0.598	0.601 (0.564,0.638)	0.598
200	15	Equal	0.599 (0.559,0.634)	0.597	0.639 (0.607,0.672)	0.639	0.601 (0.561,0.636)	0.599	0.601 (0.561,0.636)	0.599	0.601 (0.561,0.636)	0.599
25	3	Not Equal	0.632 (0.554,0.692)	0.615	0.647 (0.556,0.714)	0.631	0.632 (0.554,0.692)	0.615	0.632 (0.554,0.692)	0.615	0.632 (0.554,0.692)	0.615
25	5	Not Equal	0.633 (0.556,0.696)	0.613	0.653 (0.569,0.725)	0.636	0.635 (0.556,0.7)	0.615	0.635 (0.556,0.7)	0.615	0.634 (0.556,0.696)	0.615
25	10	Not Equal	0.63 (0.554,0.692)	0.611	0.667 (0.582,0.738)	0.653	0.627 (0.554,0.679)	0.610	0.627 (0.554,0.679)	0.610	0.63 (0.556,0.688)	0.613
25	15	Not Equal	0.629 (0.554,0.681)	0.611	0.67 (0.571,0.75)	0.661	0.628 (0.554,0.681)	0.611	0.628 (0.554,0.681)	0.611	0.626 (0.556,0.677)	0.607
100	3	Not Equal	0.596 (0.547,0.637)	0.589	0.608 (0.561,0.651)	0.604	0.596 (0.546,0.638)	0.589	0.596 (0.546,0.638)	0.589	0.596 (0.546,0.638)	0.589
100	5	Not Equal	0.596 (0.545,0.636)	0.589	0.62 (0.569,0.666)	0.615	0.597 (0.545,0.638)	0.591	0.597 (0.545,0.638)	0.591	0.597 (0.545,0.638)	0.591
100	10	Not Equal	0.593 (0.54,0.637)	0.586	0.635 (0.584,0.684)	0.635	0.596 (0.541,0.64)	0.590	0.596 (0.541,0.64)	0.590	0.596 (0.541,0.64)	0.590
100	15	Not Equal	0.589 (0.54,0.629)	0.580	0.643 (0.596,0.69)	0.644	0.592 (0.543,0.633)	0.585	0.592 (0.543,0.633)	0.585	0.592 (0.543,0.633)	0.585
200	3	Not Equal	0.596 (0.558,0.631)	0.595	0.602 (0.564,0.639)	0.602	0.596 (0.559,0.631)	0.596	0.596 (0.559,0.631)	0.596	0.596 (0.559,0.631)	0.596
200	5	Not Equal	0.601 (0.566,0.637)	0.603	0.612 (0.58,0.647)	0.611	0.602 (0.569,0.637)	0.603	0.602 (0.569,0.637)	0.603	0.602 (0.569,0.637)	0.603
200	10	Not Equal	0.605 (0.569,0.642)	0.602	0.633 (0.599,0.667)	0.634	0.607 (0.571,0.643)	0.605	0.607 (0.571,0.643)	0.605	0.607 (0.571,0.643)	0.605
200	15	Not Equal	0.606 (0.566,0.642)	0.604	0.644 (0.611,0.677)	0.643	0.608 (0.57,0.644)	0.606	0.608 (0.57,0.644)	0.606	0.608 (0.57,0.644)	0.606

Note: Statistics presented in this table were derived from test datasets described in Section 4.

Table S9. Mean AUC values with 95% confidence intervals obtained for Beta distributions in unequal allocation settings

			LogRes		MaxMutInf		MaxEnt		MinRelEnt		MinCrEnt	
**n**	**d**	**Covariance**	**Mean (95%CI)**	**Median**	**Mean (95%CI)**	**Median**	**Mean (95%CI)**	**Median**	**Mean (95%CI)**	**Median**	**Mean (95%CI)**	**Median**
25	3	Equal	0.641 (0.556,0.708)	0.624	0.662 (0.569,0.738)	0.650	0.641 (0.556,0.708)	0.625	0.641 (0.556,0.708)	0.625	0.641 (0.556,0.708)	0.625
25	5	Equal	0.642 (0.558,0.708)	0.625	0.673 (0.583,0.753)	0.662	0.643 (0.558,0.708)	0.625	0.643 (0.558,0.708)	0.625	0.644 (0.558,0.708)	0.625
25	10	Equal	0.639 (0.558,0.696)	0.623	0.688 (0.597,0.767)	0.677	0.638 (0.558,0.7)	0.615	0.638 (0.558,0.7)	0.615	0.638 (0.556,0.696)	0.622
25	15	Equal	0.635 (0.554,0.694)	0.615	0.688 (0.597,0.776)	0.677	0.639 (0.554,0.708)	0.622	0.639 (0.554,0.708)	0.622	0.636 (0.556,0.708)	0.611
100	3	Equal	0.622 (0.57,0.668)	0.621	0.633 (0.589,0.678)	0.630	0.622 (0.571,0.668)	0.621	0.622 (0.571,0.668)	0.621	0.622 (0.571,0.668)	0.621
100	5	Equal	0.625 (0.574,0.675)	0.624	0.647 (0.6,0.696)	0.644	0.626 (0.575,0.675)	0.624	0.626 (0.575,0.675)	0.624	0.626 (0.575,0.675)	0.624
100	10	Equal	0.623 (0.568,0.674)	0.619	0.666 (0.617,0.715)	0.667	0.625 (0.571,0.675)	0.623	0.625 (0.571,0.675)	0.623	0.625 (0.571,0.675)	0.623
100	15	Equal	0.615 (0.562,0.663)	0.614	0.675 (0.631,0.719)	0.675	0.618 (0.564,0.667)	0.615	0.618 (0.564,0.667)	0.615	0.618 (0.564,0.667)	0.615
200	3	Equal	0.631 (0.597,0.669)	0.631	0.629 (0.594,0.666)	0.632	0.631 (0.597,0.668)	0.631	0.631 (0.597,0.668)	0.631	0.631 (0.597,0.668)	0.631
200	5	Equal	0.638 (0.606,0.671)	0.641	0.641 (0.61,0.675)	0.643	0.638 (0.607,0.672)	0.642	0.638 (0.607,0.672)	0.642	0.638 (0.607,0.672)	0.642
200	10	Equal	0.646 (0.61,0.684)	0.645	0.665 (0.631,0.698)	0.666	0.647 (0.612,0.685)	0.647	0.647 (0.612,0.685)	0.647	0.647 (0.612,0.685)	0.647
200	15	Equal	0.648 (0.612,0.685)	0.648	0.676 (0.645,0.709)	0.676	0.649 (0.613,0.686)	0.649	0.649 (0.613,0.686)	0.649	0.649 (0.613,0.686)	0.649
25	3	Not Equal	0.642 (0.558,0.708)	0.625	0.663 (0.569,0.738)	0.649	0.642 (0.558,0.708)	0.625	0.642 (0.558,0.708)	0.625	0.643 (0.558,0.708)	0.625
25	5	Not Equal	0.644 (0.558,0.714)	0.625	0.674 (0.583,0.754)	0.662	0.646 (0.569,0.715)	0.625	0.646 (0.569,0.715)	0.625	0.647 (0.569,0.714)	0.625
25	10	Not Equal	0.639 (0.558,0.697)	0.623	0.693 (0.6,0.769)	0.681	0.639 (0.556,0.701)	0.622	0.639 (0.556,0.701)	0.622	0.639 (0.558,0.7)	0.623
25	15	Not Equal	0.635 (0.554,0.697)	0.615	0.695 (0.597,0.785)	0.681	0.641 (0.556,0.708)	0.615	0.641 (0.556,0.708)	0.615	0.638 (0.556,0.701)	0.622
100	3	Not Equal	0.624 (0.572,0.67)	0.623	0.634 (0.59,0.679)	0.631	0.625 (0.572,0.67)	0.623	0.625 (0.572,0.67)	0.623	0.625 (0.572,0.67)	0.623
100	5	Not Equal	0.63 (0.579,0.678)	0.628	0.649 (0.602,0.7)	0.646	0.631 (0.579,0.679)	0.629	0.631 (0.579,0.679)	0.629	0.631 (0.579,0.679)	0.629
100	10	Not Equal	0.631 (0.574,0.682)	0.629	0.671 (0.622,0.72)	0.672	0.633 (0.578,0.684)	0.632	0.633 (0.578,0.684)	0.632	0.633 (0.578,0.684)	0.632
100	15	Not Equal	0.624 (0.569,0.674)	0.623	0.682 (0.638,0.726)	0.684	0.627 (0.574,0.678)	0.627	0.627 (0.574,0.678)	0.627	0.627 (0.574,0.678)	0.627
200	3	Not Equal	0.633 (0.599,0.669)	0.633	0.63 (0.596,0.667)	0.633	0.633 (0.599,0.67)	0.633	0.633 (0.599,0.67)	0.633	0.633 (0.599,0.67)	0.633
200	5	Not Equal	0.642 (0.61,0.677)	0.646	0.643 (0.612,0.678)	0.646	0.643 (0.61,0.677)	0.646	0.643 (0.61,0.677)	0.646	0.643 (0.61,0.677)	0.646
200	10	Not Equal	0.654 (0.619,0.693)	0.654	0.669 (0.637,0.703)	0.671	0.656 (0.62,0.694)	0.655	0.656 (0.62,0.694)	0.655	0.656 (0.62,0.694)	0.655
200	15	Not Equal	0.658 (0.622,0.696)	0.659	0.683 (0.651,0.716)	0.683	0.66 (0.625,0.696)	0.660	0.66 (0.625,0.696)	0.660	0.66 (0.625,0.696)	0.660

Note: Statistics presented in this table were derived from test datasets described in Section 4.

Table S10. Mean AUPRC values with 95% confidence intervals obtained for multivariate Normal distributions in unequal allocation settings

			LogRes		MaxMutInf		MaxEnt		MinRelEnt		MinCrEnt	
**n**	**d**	**Covariance**	**Mean (95%CI)**	**Median**	**Mean (95%CI)**	**Median**	**Mean (95%CI)**	**Median**	**Mean (95%CI)**	**Median**	**Mean (95%CI)**	**Median**
25	3	Equal	0.387 (0.263,0.498)	0.356	0.417 (0.283,0.535)	0.395	0.399 (0.266,0.521)	0.369	0.4 (0.268,0.519)	0.370	0.399 (0.267,0.519)	0.370
25	5	Equal	0.371 (0.263,0.47)	0.353	0.407 (0.297,0.512)	0.389	0.382 (0.269,0.473)	0.361	0.385 (0.267,0.481)	0.361	0.384 (0.267,0.476)	0.360
25	10	Equal	0.333 (0.247,0.407)	0.324	0.384 (0.281,0.476)	0.364	0.338 (0.246,0.417)	0.320	0.338 (0.246,0.417)	0.322	0.334 (0.243,0.412)	0.320
25	15	Equal	0.333 (0.247,0.401)	0.315	0.389 (0.288,0.47)	0.371	0.33 (0.246,0.395)	0.316	0.33 (0.246,0.395)	0.316	0.326 (0.243,0.394)	0.310
100	3	Equal	0.427 (0.365,0.487)	0.424	0.411 (0.35,0.463)	0.404	0.429 (0.366,0.49)	0.424	0.429 (0.367,0.491)	0.424	0.429 (0.366,0.49)	0.424
100	5	Equal	0.416 (0.354,0.475)	0.410	0.409 (0.351,0.462)	0.404	0.417 (0.352,0.476)	0.412	0.417 (0.353,0.477)	0.412	0.417 (0.353,0.478)	0.412
100	10	Equal	0.417 (0.356,0.47)	0.411	0.424 (0.365,0.481)	0.420	0.421 (0.357,0.477)	0.417	0.421 (0.358,0.476)	0.416	0.42 (0.355,0.477)	0.417
100	15	Equal	0.416 (0.359,0.467)	0.410	0.428 (0.372,0.479)	0.424	0.421 (0.364,0.478)	0.413	0.422 (0.365,0.476)	0.415	0.421 (0.365,0.478)	0.414
200	3	Equal	0.43 (0.386,0.471)	0.429	0.405 (0.364,0.444)	0.403	0.431 (0.385,0.474)	0.431	0.431 (0.385,0.473)	0.432	0.431 (0.385,0.473)	0.431
200	5	Equal	0.432 (0.386,0.473)	0.431	0.411 (0.372,0.45)	0.410	0.434 (0.386,0.475)	0.432	0.434 (0.385,0.475)	0.433	0.433 (0.385,0.475)	0.432
200	10	Equal	0.445 (0.396,0.489)	0.444	0.426 (0.383,0.466)	0.422	0.446 (0.398,0.491)	0.445	0.446 (0.399,0.491)	0.446	0.446 (0.399,0.491)	0.445
200	15	Equal	0.451 (0.405,0.494)	0.449	0.432 (0.394,0.469)	0.427	0.453 (0.408,0.498)	0.452	0.452 (0.408,0.499)	0.453	0.452 (0.407,0.495)	0.452
25	3	Not Equal	0.386 (0.264,0.493)	0.359	0.42 (0.284,0.534)	0.404	0.401 (0.268,0.526)	0.373	0.4 (0.266,0.525)	0.369	0.401 (0.269,0.525)	0.372
25	5	Not Equal	0.369 (0.26,0.467)	0.348	0.416 (0.301,0.522)	0.401	0.384 (0.269,0.476)	0.361	0.384 (0.269,0.479)	0.362	0.385 (0.27,0.476)	0.362
25	10	Not Equal	0.334 (0.249,0.406)	0.328	0.39 (0.282,0.483)	0.374	0.339 (0.246,0.417)	0.320	0.339 (0.246,0.417)	0.320	0.335 (0.241,0.412)	0.318
25	15	Not Equal	0.333 (0.247,0.401)	0.314	0.4 (0.294,0.497)	0.388	0.331 (0.246,0.4)	0.316	0.331 (0.246,0.4)	0.316	0.329 (0.247,0.398)	0.312
100	3	Not Equal	0.427 (0.364,0.489)	0.423	0.416 (0.355,0.473)	0.411	0.429 (0.365,0.492)	0.426	0.429 (0.365,0.491)	0.425	0.429 (0.365,0.492)	0.426
100	5	Not Equal	0.417 (0.354,0.478)	0.413	0.419 (0.359,0.471)	0.413	0.42 (0.357,0.478)	0.416	0.42 (0.356,0.48)	0.416	0.42 (0.356,0.48)	0.415
100	10	Not Equal	0.421 (0.358,0.478)	0.416	0.442 (0.379,0.503)	0.438	0.425 (0.362,0.484)	0.420	0.425 (0.362,0.484)	0.421	0.426 (0.363,0.484)	0.423
100	15	Not Equal	0.419 (0.363,0.471)	0.413	0.452 (0.389,0.509)	0.448	0.425 (0.368,0.479)	0.418	0.425 (0.366,0.48)	0.419	0.426 (0.367,0.482)	0.420
200	3	Not Equal	0.431 (0.386,0.472)	0.429	0.412 (0.369,0.452)	0.408	0.432 (0.385,0.473)	0.431	0.432 (0.385,0.473)	0.431	0.432 (0.385,0.473)	0.431
200	5	Not Equal	0.436 (0.388,0.479)	0.436	0.423 (0.382,0.464)	0.423	0.438 (0.39,0.48)	0.437	0.438 (0.39,0.48)	0.436	0.438 (0.39,0.481)	0.437
200	10	Not Equal	0.45 (0.402,0.497)	0.448	0.448 (0.402,0.49)	0.443	0.453 (0.403,0.499)	0.452	0.453 (0.403,0.499)	0.450	0.453 (0.403,0.5)	0.451
200	15	Not Equal	0.457 (0.411,0.502)	0.456	0.459 (0.419,0.501)	0.456	0.46 (0.413,0.505)	0.458	0.46 (0.414,0.504)	0.458	0.459 (0.415,0.505)	0.458

Note: Statistics presented in this table were derived from test datasets described in Section 4.

Table S11. Mean AUPRC values with 95% confidence intervals obtained for Gamma distributions in unequal allocation settings

			LogRes		MaxMutInf		MaxEnt		MinRelEnt		MinCrEnt	
**n**	**d**	**Covariance**	**Mean (95%CI)**	**Median**	**Mean (95%CI)**	**Median**	**Mean (95%CI)**	**Median**	**Mean (95%CI)**	**Median**	**Mean (95%CI)**	**Median**
25	3	Equal	0.367 (0.265,0.452)	0.349	0.398 (0.292,0.486)	0.381	0.384 (0.272,0.483)	0.363	0.386 (0.272,0.485)	0.366	0.387 (0.272,0.49)	0.367
25	5	Equal	0.363 (0.272,0.441)	0.344	0.397 (0.295,0.489)	0.387	0.379 (0.272,0.467)	0.365	0.378 (0.272,0.466)	0.361	0.379 (0.272,0.469)	0.362
25	10	Equal	0.332 (0.242,0.403)	0.314	0.399 (0.297,0.491)	0.387	0.339 (0.247,0.409)	0.324	0.339 (0.246,0.409)	0.324	0.339 (0.246,0.414)	0.325
25	15	Equal	0.316 (0.233,0.388)	0.301	0.391 (0.286,0.486)	0.378	0.322 (0.237,0.388)	0.309	0.322 (0.237,0.388)	0.309	0.32 (0.239,0.387)	0.301
100	3	Equal	0.41 (0.35,0.464)	0.408	0.423 (0.368,0.474)	0.419	0.417 (0.354,0.473)	0.417	0.417 (0.356,0.474)	0.415	0.417 (0.354,0.473)	0.415
100	5	Equal	0.408 (0.348,0.461)	0.401	0.432 (0.374,0.487)	0.423	0.415 (0.354,0.472)	0.408	0.415 (0.355,0.473)	0.409	0.416 (0.355,0.473)	0.409
100	10	Equal	0.401 (0.339,0.453)	0.394	0.446 (0.388,0.5)	0.444	0.407 (0.343,0.461)	0.402	0.407 (0.343,0.461)	0.402	0.407 (0.344,0.461)	0.402
100	15	Equal	0.396 (0.339,0.45)	0.388	0.454 (0.392,0.508)	0.449	0.402 (0.341,0.457)	0.395	0.402 (0.342,0.458)	0.395	0.402 (0.341,0.459)	0.396
200	3	Equal	0.417 (0.375,0.454)	0.414	0.423 (0.379,0.464)	0.423	0.422 (0.377,0.461)	0.419	0.422 (0.377,0.462)	0.419	0.422 (0.376,0.461)	0.419
200	5	Equal	0.421 (0.378,0.463)	0.420	0.431 (0.389,0.473)	0.428	0.425 (0.382,0.468)	0.424	0.425 (0.381,0.467)	0.424	0.425 (0.382,0.466)	0.423
200	10	Equal	0.426 (0.383,0.47)	0.424	0.454 (0.411,0.495)	0.454	0.43 (0.385,0.474)	0.427	0.431 (0.386,0.475)	0.427	0.43 (0.384,0.474)	0.427
200	15	Equal	0.424 (0.377,0.465)	0.420	0.462 (0.42,0.502)	0.459	0.428 (0.38,0.469)	0.424	0.428 (0.38,0.47)	0.424	0.428 (0.379,0.469)	0.424
25	3	Not Equal	0.367 (0.265,0.456)	0.349	0.401 (0.292,0.497)	0.382	0.384 (0.273,0.481)	0.365	0.384 (0.272,0.483)	0.366	0.385 (0.273,0.485)	0.365
25	5	Not Equal	0.362 (0.272,0.441)	0.344	0.399 (0.295,0.494)	0.390	0.377 (0.271,0.464)	0.358	0.377 (0.272,0.464)	0.359	0.378 (0.272,0.466)	0.358
25	10	Not Equal	0.333 (0.243,0.406)	0.314	0.404 (0.298,0.501)	0.392	0.341 (0.247,0.413)	0.323	0.34 (0.247,0.413)	0.323	0.341 (0.247,0.415)	0.326
25	15	Not Equal	0.317 (0.233,0.39)	0.301	0.399 (0.291,0.499)	0.386	0.323 (0.235,0.396)	0.309	0.323 (0.235,0.396)	0.309	0.321 (0.24,0.39)	0.304
100	3	Not Equal	0.412 (0.353,0.468)	0.410	0.428 (0.371,0.481)	0.422	0.42 (0.359,0.478)	0.418	0.42 (0.357,0.478)	0.418	0.419 (0.356,0.478)	0.418
100	5	Not Equal	0.413 (0.352,0.468)	0.409	0.441 (0.382,0.496)	0.433	0.421 (0.358,0.48)	0.415	0.421 (0.359,0.474)	0.414	0.421 (0.358,0.477)	0.414
100	10	Not Equal	0.407 (0.345,0.462)	0.403	0.464 (0.404,0.522)	0.463	0.414 (0.349,0.472)	0.410	0.414 (0.349,0.471)	0.411	0.414 (0.351,0.473)	0.409
100	15	Not Equal	0.402 (0.341,0.457)	0.393	0.478 (0.413,0.537)	0.473	0.408 (0.346,0.466)	0.402	0.409 (0.347,0.469)	0.402	0.408 (0.347,0.466)	0.402
200	3	Not Equal	0.422 (0.378,0.462)	0.419	0.428 (0.382,0.471)	0.430	0.427 (0.381,0.468)	0.425	0.427 (0.381,0.468)	0.425	0.427 (0.383,0.467)	0.425
200	5	Not Equal	0.428 (0.384,0.472)	0.428	0.441 (0.397,0.484)	0.439	0.433 (0.388,0.477)	0.433	0.433 (0.388,0.477)	0.432	0.433 (0.388,0.477)	0.433
200	10	Not Equal	0.438 (0.393,0.485)	0.436	0.474 (0.428,0.518)	0.474	0.443 (0.396,0.49)	0.440	0.443 (0.396,0.49)	0.439	0.443 (0.395,0.49)	0.439
200	15	Not Equal	0.437 (0.389,0.483)	0.433	0.489 (0.445,0.533)	0.487	0.441 (0.393,0.486)	0.437	0.441 (0.394,0.486)	0.437	0.442 (0.393,0.486)	0.437

Note: Statistics presented in this table were derived from test datasets described in Section 4.

Table S12. Mean AUPRC values with 95% confidence intervals obtained for Beta distributions in unequal allocation settings

			LogRes		MaxMutInf		MaxEnt		MinRelEnt		MinCrEnt	
**n**	**d**	**Covariance**	**Mean (95%CI)**	**Median**	**Mean (95%CI)**	**Median**	**Mean (95%CI)**	**Median**	**Mean (95%CI)**	**Median**	**Mean (95%CI)**	**Median**
25	3	Equal	0.39 (0.284,0.485)	0.372	0.49 (0.347,0.621)	0.476	0.435 (0.296,0.557)	0.415	0.436 (0.296,0.558)	0.416	0.435 (0.296,0.558)	0.416
25	5	Equal	0.377 (0.278,0.455)	0.357	0.483 (0.357,0.604)	0.475	0.426 (0.296,0.531)	0.402	0.425 (0.297,0.531)	0.405	0.424 (0.296,0.53)	0.401
25	10	Equal	0.345 (0.252,0.426)	0.327	0.445 (0.32,0.559)	0.433	0.358 (0.254,0.443)	0.339	0.357 (0.254,0.442)	0.337	0.356 (0.252,0.441)	0.338
25	15	Equal	0.331 (0.238,0.406)	0.313	0.415 (0.301,0.513)	0.405	0.336 (0.24,0.414)	0.322	0.336 (0.24,0.414)	0.322	0.333 (0.24,0.408)	0.316
100	3	Equal	0.443 (0.383,0.499)	0.441	0.466 (0.407,0.524)	0.460	0.454 (0.389,0.516)	0.452	0.454 (0.389,0.516)	0.452	0.454 (0.389,0.516)	0.452
100	5	Equal	0.443 (0.381,0.499)	0.437	0.473 (0.407,0.536)	0.466	0.459 (0.39,0.519)	0.451	0.458 (0.39,0.519)	0.450	0.458 (0.39,0.519)	0.449
100	10	Equal	0.432 (0.367,0.495)	0.426	0.48 (0.418,0.538)	0.476	0.454 (0.383,0.519)	0.452	0.454 (0.384,0.518)	0.451	0.454 (0.383,0.518)	0.452
100	15	Equal	0.424 (0.365,0.48)	0.416	0.488 (0.426,0.55)	0.482	0.441 (0.376,0.503)	0.437	0.441 (0.376,0.501)	0.435	0.442 (0.376,0.504)	0.436
200	3	Equal	0.453 (0.408,0.497)	0.450	0.457 (0.41,0.499)	0.457	0.458 (0.412,0.503)	0.454	0.458 (0.412,0.503)	0.454	0.458 (0.412,0.503)	0.454
200	5	Equal	0.457 (0.411,0.501)	0.456	0.464 (0.423,0.509)	0.461	0.464 (0.418,0.51)	0.463	0.464 (0.418,0.51)	0.463	0.464 (0.418,0.51)	0.463
200	10	Equal	0.468 (0.42,0.513)	0.465	0.487 (0.44,0.531)	0.486	0.48 (0.43,0.526)	0.478	0.48 (0.431,0.526)	0.478	0.48 (0.431,0.526)	0.477
200	15	Equal	0.466 (0.419,0.511)	0.461	0.497 (0.453,0.537)	0.494	0.478 (0.427,0.526)	0.474	0.478 (0.427,0.525)	0.474	0.478 (0.428,0.527)	0.474
25	3	Not Equal	0.391 (0.283,0.484)	0.374	0.495 (0.352,0.626)	0.484	0.438 (0.298,0.559)	0.421	0.437 (0.298,0.559)	0.420	0.438 (0.298,0.56)	0.420
25	5	Not Equal	0.379 (0.278,0.456)	0.360	0.493 (0.358,0.623)	0.479	0.427 (0.298,0.529)	0.408	0.427 (0.296,0.53)	0.407	0.427 (0.294,0.532)	0.405
25	10	Not Equal	0.345 (0.251,0.424)	0.327	0.46 (0.33,0.581)	0.450	0.36 (0.256,0.442)	0.338	0.36 (0.256,0.443)	0.340	0.357 (0.253,0.445)	0.339
25	15	Not Equal	0.33 (0.238,0.403)	0.311	0.426 (0.31,0.528)	0.420	0.337 (0.243,0.415)	0.318	0.337 (0.243,0.415)	0.318	0.332 (0.243,0.403)	0.317
100	3	Not Equal	0.449 (0.389,0.507)	0.445	0.475 (0.415,0.533)	0.470	0.462 (0.397,0.526)	0.459	0.462 (0.397,0.526)	0.459	0.462 (0.397,0.526)	0.459
100	5	Not Equal	0.453 (0.388,0.508)	0.446	0.488 (0.42,0.547)	0.480	0.47 (0.4,0.533)	0.464	0.47 (0.4,0.533)	0.464	0.47 (0.4,0.534)	0.464
100	10	Not Equal	0.446 (0.377,0.504)	0.442	0.504 (0.438,0.569)	0.502	0.468 (0.398,0.535)	0.465	0.469 (0.398,0.536)	0.467	0.469 (0.398,0.536)	0.465
100	15	Not Equal	0.437 (0.375,0.497)	0.432	0.518 (0.456,0.581)	0.511	0.456 (0.386,0.519)	0.447	0.456 (0.389,0.518)	0.451	0.456 (0.391,0.519)	0.451
200	3	Not Equal	0.461 (0.415,0.506)	0.457	0.466 (0.419,0.51)	0.468	0.467 (0.42,0.515)	0.464	0.467 (0.42,0.515)	0.464	0.467 (0.42,0.515)	0.464
200	5	Not Equal	0.47 (0.422,0.514)	0.470	0.479 (0.436,0.524)	0.477	0.479 (0.429,0.527)	0.479	0.479 (0.429,0.527)	0.479	0.479 (0.429,0.527)	0.479
200	10	Not Equal	0.487 (0.439,0.534)	0.486	0.513 (0.465,0.56)	0.514	0.501 (0.449,0.551)	0.501	0.501 (0.449,0.55)	0.502	0.501 (0.449,0.55)	0.502
200	15	Not Equal	0.488 (0.439,0.535)	0.486	0.53 (0.487,0.572)	0.528	0.502 (0.45,0.552)	0.499	0.502 (0.45,0.551)	0.499	0.502 (0.45,0.551)	0.500

Note: Statistics presented in this table were derived from test datasets described in Section 4.

Table S13. Mean MCC values with 95% confidence intervals obtained for multivarite Normal distributions in unequal allocation settings

			LogRes		MaxMutInf		MaxEnt		MinRelEnt		MinCrEnt	
**n**	**d**	**Covariance**	**Mean (95%CI)**	**Median**	**Mean (95%CI)**	**Median**	**Mean (95%CI)**	**Median**	**Mean (95%CI)**	**Median**	**Mean (95%CI)**	**Median**
25	3	Equal	0.06 (-0.124,0.25)	0.081	0.094 (-0.094,0.255)	0.124	0.06 (-0.125,0.25)	0.081	0.06 (-0.125,0.25)	0.081	0.06 (-0.125,0.25)	0.081
25	5	Equal	0.06 (-0.15,0.255)	0.081	0.112 (-0.055,0.277)	0.149	0.055 (-0.125,0.25)	0.080	0.055 (-0.125,0.25)	0.080	0.057 (-0.125,0.25)	0.081
25	10	Equal	0.024 (-0.158,0.204)	0.014	0.118 (-0.055,0.269)	0.149	0.042 (-0.15,0.209)	0.055	0.042 (-0.15,0.209)	0.055	0.03 (-0.155,0.209)	0.055
25	15	Equal	0.018 (-0.175,0.194)	0.033	0.14 (-0.014,0.316)	0.169	0.021 (-0.171,0.194)	0.033	0.021 (-0.171,0.194)	0.033	0.018 (-0.158,0.19)	0.014
100	3	Equal	0.106 (0.028,0.189)	0.110	0.088 (0.011,0.166)	0.095	0.106 (0.025,0.186)	0.108	0.106 (0.025,0.186)	0.108	0.106 (0.025,0.186)	0.108
100	5	Equal	0.097 (0.02,0.176)	0.099	0.098 (0.021,0.178)	0.098	0.1 (0.019,0.179)	0.104	0.1 (0.019,0.179)	0.104	0.1 (0.019,0.179)	0.104
100	10	Equal	0.109 (0.026,0.199)	0.111	0.126 (0.053,0.205)	0.122	0.112 (0.034,0.2)	0.115	0.112 (0.034,0.2)	0.115	0.112 (0.034,0.2)	0.115
100	15	Equal	0.112 (0.038,0.193)	0.113	0.134 (0.063,0.208)	0.139	0.117 (0.029,0.196)	0.124	0.117 (0.029,0.196)	0.124	0.117 (0.029,0.196)	0.124
200	3	Equal	0.114 (0.057,0.171)	0.119	0.092 (0.038,0.152)	0.096	0.116 (0.061,0.168)	0.117	0.116 (0.061,0.168)	0.117	0.116 (0.061,0.168)	0.117
200	5	Equal	0.119 (0.062,0.175)	0.121	0.104 (0.051,0.16)	0.107	0.118 (0.06,0.177)	0.123	0.118 (0.06,0.177)	0.123	0.118 (0.06,0.177)	0.123
200	10	Equal	0.141 (0.081,0.199)	0.139	0.121 (0.067,0.177)	0.121	0.139 (0.077,0.198)	0.141	0.139 (0.077,0.198)	0.141	0.139 (0.077,0.198)	0.141
200	15	Equal	0.144 (0.087,0.199)	0.143	0.132 (0.075,0.186)	0.130	0.146 (0.086,0.206)	0.148	0.146 (0.086,0.206)	0.148	0.146 (0.086,0.206)	0.148
25	3	Not Equal	0.062 (-0.12,0.25)	0.081	0.093 (-0.088,0.255)	0.124	0.061 (-0.125,0.241)	0.081	0.061 (-0.125,0.241)	0.081	0.061 (-0.125,0.241)	0.081
25	5	Not Equal	0.064 (-0.125,0.25)	0.088	0.11 (-0.055,0.271)	0.124	0.065 (-0.123,0.25)	0.088	0.065 (-0.123,0.25)	0.088	0.066 (-0.124,0.25)	0.088
25	10	Not Equal	0.033 (-0.152,0.2)	0.055	0.124 (-0.033,0.277)	0.150	0.044 (-0.13,0.219)	0.055	0.044 (-0.13,0.219)	0.055	0.035 (-0.158,0.209)	0.055
25	15	Not Equal	0.035 (-0.158,0.207)	0.055	0.149 (0,0.322)	0.175	0.031 (-0.169,0.2)	0.055	0.031 (-0.169,0.2)	0.055	0.025 (-0.158,0.2)	0.029
100	3	Not Equal	0.107 (0.028,0.19)	0.117	0.088 (0.011,0.166)	0.087	0.109 (0.028,0.19)	0.119	0.109 (0.028,0.19)	0.119	0.109 (0.028,0.19)	0.119
100	5	Not Equal	0.097 (0.016,0.184)	0.099	0.104 (0.03,0.186)	0.104	0.1 (0.02,0.182)	0.100	0.1 (0.02,0.182)	0.100	0.1 (0.02,0.182)	0.100
100	10	Not Equal	0.115 (0.038,0.194)	0.119	0.127 (0.05,0.21)	0.127	0.115 (0.041,0.199)	0.122	0.115 (0.041,0.199)	0.122	0.115 (0.041,0.199)	0.122
100	15	Not Equal	0.116 (0.04,0.198)	0.116	0.14 (0.066,0.217)	0.141	0.115 (0.038,0.195)	0.119	0.115 (0.038,0.195)	0.119	0.115 (0.038,0.195)	0.119
200	3	Not Equal	0.115 (0.056,0.175)	0.118	0.092 (0.041,0.149)	0.096	0.116 (0.06,0.173)	0.121	0.116 (0.06,0.173)	0.121	0.116 (0.06,0.173)	0.121
200	5	Not Equal	0.124 (0.069,0.182)	0.125	0.109 (0.056,0.167)	0.111	0.123 (0.064,0.185)	0.120	0.123 (0.064,0.185)	0.120	0.123 (0.064,0.185)	0.120
200	10	Not Equal	0.142 (0.082,0.201)	0.141	0.129 (0.073,0.186)	0.126	0.142 (0.09,0.202)	0.141	0.142 (0.09,0.202)	0.141	0.142 (0.09,0.202)	0.141
200	15	Not Equal	0.152 (0.097,0.211)	0.151	0.14 (0.08,0.198)	0.141	0.156 (0.095,0.217)	0.162	0.156 (0.095,0.217)	0.162	0.156 (0.095,0.217)	0.162

Note: Statistics presented in this table were derived from test datasets described in Section 4.

Table S14. Mean MCC values with 95% confidence intervals obtained for Gamma distributions in unequal allocation settings

			LogRes		MaxMutInf		MaxEnt		MinRelEnt		MinCrEnt	
**n**	**d**	**Covariance**	**Mean (95%CI)**	**Median**	**Mean (95%CI)**	**Median**	**Mean (95%CI)**	**Median**	**Mean (95%CI)**	**Median**	**Mean (95%CI)**	**Median**
25	3	Equal	0.046 (-0.127,0.239)	0.065	0.121 (-0.055,0.304)	0.125	0.047 (-0.15,0.236)	0.065	0.047 (-0.15,0.236)	0.065	0.047 (-0.15,0.236)	0.065
25	5	Equal	0.058 (-0.125,0.24)	0.081	0.136 (-0.025,0.305)	0.155	0.057 (-0.125,0.24)	0.065	0.057 (-0.125,0.24)	0.065	0.054 (-0.125,0.239)	0.060
25	10	Equal	0.032 (-0.158,0.219)	0.051	0.146 (-0.014,0.316)	0.161	0.034 (-0.158,0.204)	0.040	0.034 (-0.158,0.204)	0.040	0.036 (-0.15,0.217)	0.055
25	15	Equal	0.01 (-0.17,0.189)	0.000	0.146 (-0.033,0.316)	0.171	0.023 (-0.161,0.204)	0.033	0.023 (-0.161,0.204)	0.033	0.017 (-0.161,0.189)	0.014
100	3	Equal	0.088 (0.01,0.173)	0.096	0.12 (0.046,0.194)	0.122	0.087 (0.002,0.173)	0.097	0.087 (0.002,0.173)	0.097	0.087 (0.002,0.173)	0.097
100	5	Equal	0.09 (0.014,0.173)	0.093	0.13 (0.055,0.208)	0.133	0.094 (0.017,0.17)	0.099	0.094 (0.017,0.17)	0.099	0.094 (0.017,0.17)	0.099
100	10	Equal	0.087 (0.008,0.17)	0.091	0.147 (0.072,0.228)	0.149	0.092 (0.012,0.177)	0.094	0.092 (0.012,0.177)	0.094	0.092 (0.012,0.177)	0.094
100	15	Equal	0.079 (0,0.163)	0.081	0.154 (0.077,0.23)	0.160	0.087 (0.001,0.169)	0.089	0.087 (0.001,0.169)	0.089	0.087 (0.001,0.169)	0.089
200	3	Equal	0.106 (0.047,0.17)	0.110	0.114 (0.056,0.176)	0.113	0.107 (0.051,0.166)	0.111	0.107 (0.051,0.166)	0.111	0.107 (0.051,0.166)	0.111
200	5	Equal	0.108 (0.049,0.168)	0.110	0.123 (0.069,0.181)	0.126	0.109 (0.053,0.168)	0.111	0.109 (0.053,0.168)	0.111	0.109 (0.053,0.168)	0.111
200	10	Equal	0.114 (0.057,0.177)	0.116	0.146 (0.087,0.206)	0.144	0.116 (0.055,0.175)	0.114	0.116 (0.055,0.175)	0.114	0.116 (0.055,0.175)	0.114
200	15	Equal	0.109 (0.045,0.173)	0.111	0.157 (0.099,0.217)	0.158	0.112 (0.051,0.172)	0.110	0.112 (0.051,0.172)	0.110	0.112 (0.051,0.172)	0.110
25	3	Not Equal	0.046 (-0.158,0.239)	0.079	0.124 (-0.051,0.299)	0.150	0.051 (-0.13,0.239)	0.079	0.051 (-0.13,0.239)	0.079	0.051 (-0.13,0.239)	0.079
25	5	Not Equal	0.062 (-0.15,0.25)	0.081	0.134 (-0.036,0.304)	0.158	0.055 (-0.15,0.236)	0.081	0.055 (-0.15,0.236)	0.081	0.051 (-0.15,0.236)	0.081
25	10	Not Equal	0.027 (-0.169,0.2)	0.033	0.15 (-0.014,0.322)	0.161	0.034 (-0.155,0.217)	0.040	0.034 (-0.155,0.217)	0.040	0.037 (-0.152,0.219)	0.051
25	15	Not Equal	0.013 (-0.171,0.189)	0.000	0.154 (-0.033,0.329)	0.171	0.021 (-0.17,0.204)	0.033	0.021 (-0.17,0.204)	0.033	0.026 (-0.152,0.194)	0.033
100	3	Not Equal	0.091 (0.002,0.183)	0.097	0.119 (0.044,0.199)	0.122	0.094 (0.007,0.183)	0.101	0.094 (0.007,0.183)	0.101	0.094 (0.007,0.183)	0.101
100	5	Not Equal	0.096 (0.019,0.178)	0.100	0.132 (0.053,0.221)	0.132	0.097 (0.01,0.182)	0.100	0.097 (0.01,0.182)	0.100	0.097 (0.01,0.182)	0.100
100	10	Not Equal	0.091 (0.008,0.174)	0.096	0.15 (0.074,0.23)	0.156	0.097 (0.017,0.177)	0.097	0.097 (0.017,0.177)	0.097	0.097 (0.017,0.177)	0.097
100	15	Not Equal	0.085 (0.008,0.165)	0.090	0.163 (0.083,0.249)	0.162	0.088 (0.005,0.173)	0.095	0.088 (0.005,0.173)	0.095	0.088 (0.005,0.173)	0.095
200	3	Not Equal	0.105 (0.049,0.165)	0.110	0.117 (0.062,0.175)	0.115	0.108 (0.052,0.171)	0.111	0.108 (0.052,0.171)	0.111	0.108 (0.052,0.171)	0.111
200	5	Not Equal	0.115 (0.056,0.171)	0.120	0.128 (0.072,0.187)	0.126	0.111 (0.052,0.176)	0.115	0.111 (0.052,0.176)	0.115	0.111 (0.052,0.176)	0.115
200	10	Not Equal	0.118 (0.059,0.182)	0.116	0.153 (0.094,0.213)	0.153	0.123 (0.062,0.184)	0.124	0.123 (0.062,0.184)	0.124	0.123 (0.062,0.184)	0.124
200	15	Not Equal	0.119 (0.06,0.183)	0.117	0.163 (0.099,0.225)	0.164	0.119 (0.056,0.177)	0.121	0.119 (0.056,0.177)	0.121	0.119 (0.056,0.177)	0.121

Note: Statistics presented in this table were derived from test datasets described in Section 4.

Table S15. Mean MCC values with 95% confidence intervals obtained for Beta distributions in unequal allocation settings

			LogRes		MaxMutInf		MaxEnt		MinRelEnt		MinCrEnt	
**n**	**d**	**Covariance**	**Mean (95%CI)**	**Median**	**Mean (95%CI)**	**Median**	**Mean (95%CI)**	**Median**	**Mean (95%CI)**	**Median**	**Mean (95%CI)**	**Median**
25	3	Equal	0.086 (-0.108,0.267)	0.114	0.153 (0,0.316)	0.175	0.083 (-0.113,0.26)	0.114	0.083 (-0.113,0.26)	0.114	0.082 (-0.113,0.26)	0.114
25	5	Equal	0.068 (-0.124,0.255)	0.086	0.189 (0.055,0.34)	0.194	0.078 (-0.106,0.254)	0.088	0.078 (-0.106,0.254)	0.088	0.079 (-0.1,0.255)	0.088
25	10	Equal	0.057 (-0.125,0.239)	0.081	0.19 (0.051,0.342)	0.200	0.067 (-0.122,0.24)	0.081	0.067 (-0.122,0.24)	0.081	0.064 (-0.126,0.25)	0.081
25	15	Equal	0.035 (-0.152,0.236)	0.036	0.198 (0.055,0.372)	0.204	0.03 (-0.171,0.219)	0.055	0.03 (-0.171,0.219)	0.055	0.035 (-0.161,0.227)	0.055
100	3	Equal	0.129 (0.053,0.212)	0.133	0.15 (0.077,0.223)	0.153	0.13 (0.054,0.212)	0.134	0.13 (0.054,0.212)	0.134	0.13 (0.054,0.212)	0.134
100	5	Equal	0.138 (0.061,0.217)	0.143	0.169 (0.1,0.243)	0.173	0.137 (0.063,0.218)	0.145	0.137 (0.063,0.218)	0.145	0.137 (0.063,0.218)	0.145
100	10	Equal	0.133 (0.051,0.221)	0.128	0.189 (0.111,0.266)	0.189	0.14 (0.054,0.227)	0.140	0.14 (0.054,0.227)	0.140	0.14 (0.054,0.227)	0.140
100	15	Equal	0.127 (0.046,0.211)	0.133	0.199 (0.121,0.271)	0.200	0.13 (0.055,0.212)	0.139	0.13 (0.055,0.212)	0.139	0.13 (0.055,0.212)	0.139
200	3	Equal	0.15 (0.094,0.208)	0.156	0.147 (0.089,0.21)	0.146	0.15 (0.09,0.212)	0.155	0.15 (0.09,0.212)	0.155	0.15 (0.09,0.212)	0.155
200	5	Equal	0.161 (0.109,0.218)	0.161	0.16 (0.103,0.218)	0.157	0.156 (0.104,0.214)	0.156	0.156 (0.104,0.214)	0.156	0.156 (0.104,0.214)	0.156
200	10	Equal	0.165 (0.105,0.229)	0.163	0.185 (0.129,0.244)	0.184	0.166 (0.107,0.228)	0.163	0.166 (0.107,0.228)	0.163	0.166 (0.107,0.228)	0.163
200	15	Equal	0.168 (0.106,0.233)	0.171	0.198 (0.135,0.261)	0.201	0.164 (0.104,0.225)	0.162	0.164 (0.104,0.225)	0.162	0.164 (0.104,0.225)	0.162
25	3	Not Equal	0.084 (-0.108,0.265)	0.122	0.161 (0,0.322)	0.189	0.084 (-0.1,0.265)	0.122	0.084 (-0.1,0.265)	0.122	0.085 (-0.1,0.265)	0.122
25	5	Not Equal	0.082 (-0.108,0.269)	0.088	0.187 (0.033,0.342)	0.204	0.084 (-0.108,0.265)	0.088	0.084 (-0.108,0.265)	0.088	0.086 (-0.1,0.265)	0.094
25	10	Not Equal	0.057 (-0.125,0.25)	0.081	0.185 (0.036,0.343)	0.193	0.067 (-0.12,0.25)	0.081	0.067 (-0.12,0.25)	0.081	0.068 (-0.124,0.25)	0.081
25	15	Not Equal	0.032 (-0.152,0.209)	0.033	0.207 (0.055,0.378)	0.224	0.032 (-0.169,0.2)	0.055	0.032 (-0.169,0.2)	0.055	0.035 (-0.152,0.221)	0.055
100	3	Not Equal	0.139 (0.061,0.221)	0.141	0.149 (0.074,0.227)	0.150	0.136 (0.059,0.22)	0.140	0.136 (0.059,0.22)	0.140	0.136 (0.059,0.22)	0.140
100	5	Not Equal	0.145 (0.068,0.223)	0.151	0.171 (0.094,0.245)	0.169	0.142 (0.069,0.214)	0.148	0.142 (0.069,0.214)	0.148	0.142 (0.069,0.214)	0.148
100	10	Not Equal	0.143 (0.063,0.224)	0.145	0.197 (0.115,0.277)	0.202	0.15 (0.067,0.233)	0.151	0.15 (0.067,0.233)	0.151	0.15 (0.067,0.233)	0.151
100	15	Not Equal	0.134 (0.046,0.225)	0.136	0.207 (0.125,0.291)	0.208	0.141 (0.063,0.226)	0.140	0.141 (0.063,0.226)	0.140	0.141 (0.063,0.226)	0.140
200	3	Not Equal	0.152 (0.093,0.211)	0.151	0.147 (0.089,0.209)	0.146	0.156 (0.096,0.214)	0.155	0.156 (0.096,0.214)	0.155	0.156 (0.096,0.214)	0.155
200	5	Not Equal	0.162 (0.104,0.222)	0.163	0.162 (0.103,0.223)	0.162	0.162 (0.099,0.229)	0.164	0.162 (0.099,0.229)	0.164	0.162 (0.099,0.229)	0.164
200	10	Not Equal	0.177 (0.114,0.237)	0.179	0.19 (0.124,0.259)	0.193	0.181 (0.115,0.244)	0.176	0.181 (0.115,0.244)	0.176	0.181 (0.115,0.244)	0.176
200	15	Not Equal	0.18 (0.115,0.249)	0.182	0.206 (0.14,0.273)	0.207	0.179 (0.117,0.242)	0.176	0.179 (0.117,0.242)	0.176	0.179 (0.117,0.242)	0.176

Note: Statistics presented in this table were derived from test datasets described in Section 4.
